# Ecological impacts of the industrial revolution in a lowland raised peat bog near Manchester, NW England

**DOI:** 10.1002/ece3.9807

**Published:** 2023-02-14

**Authors:** Sandra Garcés‐Pastor, William J. Fletcher, Peter A. Ryan

**Affiliations:** ^1^ Quaternary Environments and Geoarchaeology Research Group, Department of Geography, School of Environment, Education and Development University of Manchester Manchester UK; ^2^ Department of Evolutionary Biology, Ecology and Environmental Sciences Universitat de Barcelona Barcelona Spain

**Keywords:** human impact, industrial revolution, paleoecology, peat bogs, pollen, UK

## Abstract

(1) Ombrotrophic peat bogs provide valuable records of environmental change on long timescales but are rarely preserved near the major centers of industrial activity. Holcroft Moss is a rare example of a stratigraphically intact lowland peat bog in NW England, which provides a valuable opportunity to trace industrial impacts on vegetation in a sensitive environmental archive close to the early industrializing cities of Manchester and Liverpool. (2) We reconstructed environmental changes at Holcroft Moss before and after the Industrial Revolution using a decadal‐scale record of pollen, non‐pollen palynomorphs, microcharcoal, peat composition (organic content and ash‐free bulk density) and heavy metal content, constrained by a radiocarbon and SCP (spheroidal carbonaceous particle) chronology. We examine the relationship between abiotic and biotic environmental tracers using principal component analysis and evaluate the role of local and regional climatic and anthropogenic drivers using canonical redundancy analysis and partitioning of variation. (3) Results show significant changes in bog vegetation composition during the last 700 years. Prior to 1750 CE, climate and agro‐pastoral activity (grazing and fires) were the main drivers of vegetation change. Subsequently, regional coal‐fired industry contributed to major increases in atmospheric pollutants (dust, heavy metals, and acid deposition) that severely impacted vegetation, driving the decline of *Sphagnum*. Grasses rose to dominance in the 20th century associated especially with bog conversion and cumulative nitrogen deposition. Although atmospheric pollution significantly decreased in the post‐industrial era, vegetation has not returned to pre‐industrial conditions, reflecting the ongoing impact of global change drivers which pose challenges for conservation and restoration. (4) *Synthesis*. Paleoecological studies are needed to reveal the long‐term history of vegetation degradation and to offer guidelines for restoration and conservation practices. This study reconstructs the last 700 years of a peat bog located between Manchester and Liverpool, revealing the timing and nature of vegetation changes across the trajectory of early industrialization and eventual post‐industrial decline. Our study reveals the progressive dominance of regional anthropogenic forcing and highlights that the present‐day vegetation does not have past analogs within the last 700 years. Conservation measures favoring the reintroduction of *Sphagnum* are justified in redressing the major biological legacy of the Industrial Revolution, while steps to increase *Calluna* should also be considered in light of its resilience to dry and fire‐prone conditions.

## Graphical abstract

This study presents the last 700 years of a peat bog located between Manchester and Liverpool, revealing the timing and nature of vegetation changes across the trajectory of early industrialization and eventual post‐industrial decline.

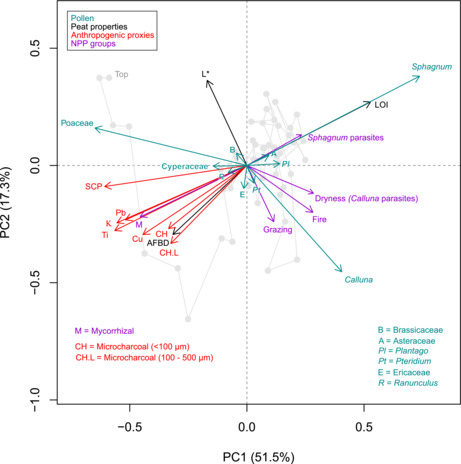

## 1 INTRODUCTION

The domination of the Earth's ecosystems by human activity is a defining feature of the contemporary world (Vitousek et al., 1997). Since the timescales of human‐driven landscape change may exceed historical ecological monitoring, paleoecological approaches are required for understanding the origins and timing of anthropogenic ecological change (Chambers et al., 2017). NW England, including the cities of Manchester and Liverpool, is often credited as “the cradle of the world's first industrial revolution” (Walton, 1987:7). The region is thus a globally relevant case study for examining the environmental impact of a centuries' long trajectory of industrialization as well as subsequent post‐industrial decline. While the legacy of industrialization has been examined in the region's soils and river sediments (e.g., Enya et al., 2019; Hurley et al., 2017), there are few continuous paleoecological records spanning this critical time period in detail. In the surrounding upland regions, atmospheric pollution and grazing have been identified as drivers of peatland degradation and erosion (e.g. Mackay & Tallis, 1996; Tallis, 1985, 1987). However, knowledge of environmental responses to human pressure in lowland landscapes in NW England is less well‐developed. This study therefore presents a new high‐resolution record of environmental change in a lowland raised peat bog spanning the multi‐centennial timescale of NW England's industrial transformation.

**FIGURE 1:**
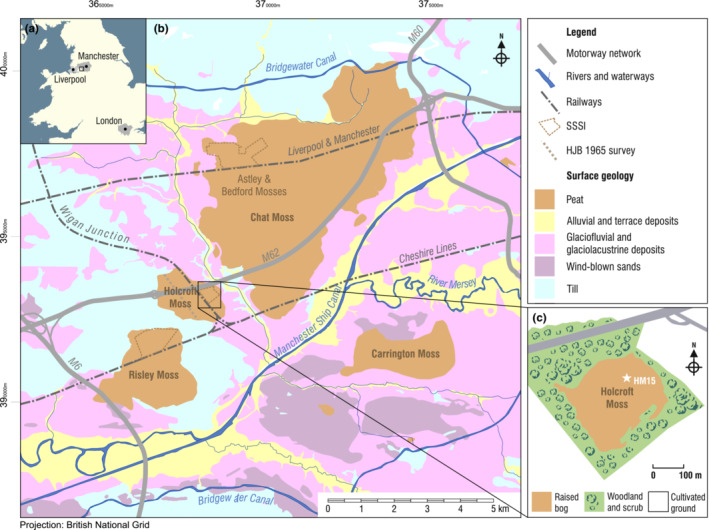
Location of the Holcroft Moss study site in NW England, showing (a) proximity to the early industrial cities of Manchester and Liverpool; (b) extent of lowland peatlands in the Greater Manchester region including Sites of Special Scientific Interest (SSSI) and the 1965 peat stratigraphical survey profile of Holcroft Moss (Birks, 1965); (c) current extent of Holcroft Moss and the location where core HM15 (white star) was retrieved. Surface geology based on DiGMapGB‐50 (Geological Map Data BGS © UKRI 2022).

Throughout history, UK peatlands have been heavily impacted by industrial activities, land management, and peat extraction. Since 1850, the total area of lowland raised bog in the UK has fallen by 94%. In NW England, 99% of the lowland peat bog habitat has been destroyed (Walker et al., 2016). The lack of peat bogs that cover the industrial time period is a drawback in many of the peatlands in the UK (McCarroll et al., 2016, 2017). Holcroft Moss in North Cheshire (historically South Lancashire) is a rare example of a stratigraphically intact fragment of a larger ombrotrophic lowland bog that was not disturbed by cutting for peat (Fletcher & Ryan, 2018). Indeed, this bog is located in the heartland of the Industrial Revolution in NW England, within 16 km of the urban areas of Manchester and Liverpool, the first industrial cities of the modern world, as well in close proximity to some of the country's earliest railway and canal infrastructure (Figure 1). The Industrial Revolution led by textile manufacturing, iron founding, and steam power had profound impacts on work and society (Chapman, 1990), but suitable natural archives to trace the associated ecological transformation are rare. Unlike many developing areas of the world, the decline of industrial activity since the mid‐20th century in NW England furthermore provides a valuable testbed for understanding the potential for recovery of natural systems (Valentine et al., 2013). Holcroft Moss therefore offers the opportunity to assess ecological impacts before and after the Industrial Revolution, providing necessary long‐term ecological knowledge for responsible peatland management (Chambers et al., 1999; McCarroll et al., 2016).

In addition to historical degradation, peatland ecosystems are threatened by global change drivers including atmospheric nitrogen deposition, rising temperatures, and drought (Fenner & Freeman, 2011; Payne, 2014). These drivers impact upon the trophic and hydrological status of peatlands, contributing to vascular plant invasion and biodiversity loss in nutrient‐poor systems as well as accelerating carbon loss (Lamers et al., 2000; Walker et al., 2016). In the UK, these global change drivers have been implicated in the increased dominance of graminoid plants, notably *Molinia caerulea* (purple moor grass), in both blanket and raised bogs (Chambers et al., 1999). However, disentangling the role of historical and current drivers of change and discriminating macroscale drivers from local disturbance impacts such as fire are challenging.

Paleoecological records with multiple tracers of past environmental changes (multi‐proxy studies) can help address the history of vegetational changes in peatlands over longer timeframes than documentary records. As such, they provide valuable site‐specific information on past environmental conditions relevant for the determination of restoration trajectories, as well as revealing the sensitivity and resilience of the studied systems (Froyd & Willis, 2008). The Manchester Mosses are the current focus of restoration activities to improve biodiversity and carbon capture, within a wider set of efforts to improve landscape connectivity and agricultural practices (Keightley, 2020; Thomas & England, 2016). New insights into historical conditions and the trajectory and drivers of change at Holcroft Moss are therefore timely and relevant to inform conservation actions.

Using paleoecological tools including analysis of pollen, non‐pollen palynomorphs (NPPs), microcharcoal, spheroidal carbonaceous particles (SCPs), peat composition (organic content and ash‐free bulk density), and heavy metal content, we aim to document vegetation response to past climate changes, disturbance events, and anthropic pressure. We perform a high‐resolution (decadal‐scale) reconstruction to understand the nature of environmental changes at Holcroft Moss during the historical era and evaluate the role of local to regional anthropogenic impacts. Furthermore, we seek to evaluate the relative contribution of local disturbance factors (grazing and fire), regional industrial impacts (atmospheric deposition of heavy metals, ash, and dust), nitrogen loading, climate change (temperature and atmospheric variability), and bog conversion.

## 2 MATERIALS AND METHODS

### 2.1 Study site

Holcroft Moss is a nature reserve located in the River Mersey catchment at 21 m above sea level in NW England, within 16 km of the urban areas of Manchester and Liverpool (Figure 1a). The site represents a fragment (19 ha) of a formerly more extensive lowland raised bog that has been reduced in areal extent by agricultural reclamation and the construction of the adjacent M62 motorway. In 1845, Holcroft Moss still extended to 185 ha (Bragg et al., 1984). By the 1890s, the area of Holcroft Moss had been reduced by ~50% by conversion to fields around the southern margins, expansion of the Holcroft First woodland area in the NW, and drain cutting. The bog was also dissected NW–SE by the construction of the Wigan Junction Railway (Reade, 1878), built to connect the Wigan collieries to Manchester with seven trains daily, which eventually closed in 1969. From the 1920s to the 1950s, historical maps show that the larger remaining western sector of the bog was mainly given over to peat‐cutting, with a pair of tramways constructed by the 1950s to facilitate export. By the 1980s, the bog had been further dissected W–E by the construction of the M62 motorway. A small fragment in the eastern sector is thus the only area to retain a full stratigraphic record of the bog's formation.

The Holocene peat stratigraphy and pollen record of Holcroft Moss was first studied by Birks (1965), when the extension of the peat bog was still considerably larger (Figure 1b). Peat overlies glacial till and glaciolacustrine sediments and reaches a maximum thickness of around 5 m. The bog vegetation at present is composed of sedges (*Eriophorum* spp.), heaths (*Erica tetralix* and *Vaccinium oxycoccus*), and several species of *Sphagnum* (*S*. *palustre*, *S*. *fallax*, and *S*. *fimbriatum*). Purple moor grass (*Molinia caerulea*) is very abundant. The margins of the bog have woodland and scrub with *Betula pubescens* and *Salix* sp. along with *Pteridium aquilinum* in the understorey (Figure 1c).

Holcroft Moss is a Natural England Site of Special Scientific Interest (SSSI) and forms part of the Manchester Mosses Special Area of Conservation (SAC). The site has been managed by the Cheshire Wildlife Trust since 1990. The conservation status is described as “unfavorable‐recovering” (Natural England, 2021). This status relates to a low current diversity of *Sphagnum* species but also reflects improvements that have been made and the implied potential for further recovery over time. The recent discovery of two nationally rare species of jumping spiders (Salticidae) highlights the importance of the site for invertebrate biodiversity (Burkmar & Gallon, 2019). The site has significant potential value as a nucleus of biodiversity in future efforts to improve connectivity across the Manchester Mosses (Figure 1).

Current conservation measures at the site include access control via a perimeter fence, Hebridean sheep grazing to reduce the dominance of *Molinia caerulea* and the encroachment of woody taxa, tree cutting, and moisture retention with the installation of a plastic pile edging and blocking of perimeter ditches (Valentine et al., 2013). Between 1998 and 2009, the site was grazed by up to 80 sheep, leading not only to a reduction in *Molinia* but also negative impacts on *Sphagnum* and other bog taxa; numbers were subsequently reduced to 18 sheep (Thomas & England, 2016). After these combined measures, about 8.6 ha has started to develop toward active bog with recovery of *Sphagnum*, *Eriophorum*, *Erica tetralix*, and *Vaccinium oxycoccus* (Thomas & England, 2016).

### 2.2 Sampling, peat characterization, and geochemical analysis

With the view to detailed study of the recent historical era, a peat surface core of 50 cm was recovered in 2015 (Figure 1c) using a Russian corer (Jowsey, 1966). The HM15 core was transported in cling‐film and plastic guttering to the Geography Laboratories of the University of Manchester. The core was carefully cleaned for visual description of peat characteristics (composition, color, elasticity, and sharpness of boundaries). The designation of stratigraphic units was guided by qualitative observation of peat components (*Sphagnum* leaves, herbaceous fragments, and humified material) under low‐power microscopy. Volume magnetic susceptibility (Κ) was measured using a Bartington Instruments MS2C core loop scanner at 1‐cm scanning intervals (Dearing, 1999). Elemental analysis was performed by x‐ray fluorescence core scanning (XRF‐CS) on a Cox Analytical ITRAX scanner, using the molybdenum tube set at 30 kV voltage, 55 mA current, 400 ms exposure time, and a 1 mm step size. Element counts for lead, copper, and titanium are presented here for their interest as tracers of heavy metal (Pb and Cu) and dust pollution (Ti), respectively. Variation in water content, organic matter content, and core density can affect the reliability of geochemical records derived by XRF‐CS on peat (Longman et al., 2019). In order to minimize these impacts, the elemental data have been normalized to the ratio of incoherent to coherent scattering (inc/coh ratio; Kylander et al., 2011), as applied, for example, in the study of anthropogenic Pb in the Füramoos peat bog in Germany (Kern et al., 2021). For Pb and Cu, where quantitative concentrations were derived by Inductively Coupled Plasma Optical Emission Spectrometry (ICP‐OES) on the same core (previously reported in Fletcher & Ryan, 2018), we find a very strong correlation between ICP‐OES and normalized XRF‐CS results (for Pb, *R* = .925, *p* < 2 × 10^−16^ and for Cu, *R* = .923, *p* < 2 × 10^−16^), giving confidence in the reliability of the XRF‐CS data.

The HM15 core displays notable visual changes in darkness, which in a peat core may typically relate to the extent of decomposition (humification). Since insufficient core material was available for destructive colorimetric humification measurement (e.g., Chambers et al., 2011) or more advanced chemical approaches (Biester et al., 2014), we consider the darkness of the peat as recorded by digital photography as an improvement over subjective visual assessment by the eye. The ITRAX incorporates a high‐quality, light‐sensitive 2048 pixel CMOS (complementary metal oxide semiconductor) device which generates a digital color (RGB) photograph of the core surface (Croudace et al., 2006). ITRAX digital photographs have previously been used to quantify sediment lightness for the characterization of sediment cores (Lee et al., 2021). Following the same methodology, the digital photograph of HM15 from the ITRAX was saved in .tiff format and cropped to exclude the irregular margins of the peat core, which would otherwise include bright background areas with no peat. The resulting .tiff image was imported into R as a raster array using the R package raster (Hijmans, 2020). The RGB color values were converted into CIE L*a*b* values with a default D65 white illumination setting, where L* is the lightness, ranging from 0 (black) to 100 (white). Median L* values for each row of pixels were calculated.

Following the non‐destructive analyses, the core was frozen and cut into 1‐cm slices using a clean knife (De Vleeschouwer & Chambers, 2010). Volumetric blocks of 1–2 cm^3^ were cut from each sub‐sample and a/b/c axes measured using electronic calipers. These were then dried at 105°C, and organic matter content was estimated by loss‐on‐ignition (LOI) at 550°C (Bengtsson & Enell, 1986). Bulk density and ash‐free bulk density (AFBD) were calculated on the basis of the dry weight and ash‐free weight of each volumetric sample, respectively (Chambers et al., 2011). In order to ensure sufficient material for pollen analysis, LOI and AFBD measurements were not obtained on the uppermost interval 0–1 cm where less material was recovered.

### 2.3 Core chronology

Three radiocarbon dates were obtained on bulk peat by the accelerator mass spectrometry (AMS) method at the Beta Analytic Radiocarbon Dating Laboratory (Miami, FL, USA). The age‐depth model was constructed on calibrated radiocarbon data and changes in bulk density (as described in Fletcher & Ryan, 2018), updating the calibration with the latest IntCal20.14C curve (Reimer et al., 2020). We improve the chronology for the last century by incorporating age estimates based on correlation of cumulative SCP abundances with the regional SCP profile for NW England following the method of Rose and Appleby (2005). We also present for comparison, but do not integrate into the HM15 age model, a fourth radiocarbon date obtained on a more recent core (HM20) recovered at the same coring location (T. Wang, unpublished PhD data).

### 2.4 Microscopic identification of pollen, non‐pollen palynomorphs, charcoal, and SCPs

A total of 50 samples were processed using standard palynological methods (Moore et al., 1991) with KOH digestion, sieving, HCl, HF, and acetolysis. Two *Lycopodium* tablets (batch 3862 and 9666 spores/tablet) were added to each sample as an exotic marker before chemical processing. Microscopic slides were mounted in glycerol and 500 main sum pollen grains were counted using a high‐powered transmitted light microscope (40× and 100× magnification). We noted two *Sphagnum* spore morphotypes according to their spore wall morphology (Tallis, 1962). Although spores do not permit identification of *Sphagnum* species to the lowest taxonomic level (Tallis, 1962), we differentiate two spore morphotypes (A, thick‐walled; B, thin walled and smaller) that show distinct trends over time and coincide with other changes in the pollen and NPP assemblages. Non‐pollen palynomorphs, sedimentary charred particles (<100 μm; 100–500 μm), and spheroidal carbonaceous particles or SCPs (>20 μm) on the same pollen slides were also counted. Data are presented as a percentage of the main pollen sum, which excludes aquatic plant pollen and spores. Due to the limitations of peat material and resources, our study focuses on microfossils (pollen and NPPs). Future investigation of plant macrofossils and/or DNA is required to test and advance the inferences drawn here from the study of microfossils.

### 2.5 Statistical analysis

Contiguous 1‐cm core sampling for all proxies (Table 1) permits the direct comparison of co‐registered proxies without chronological uncertainty. High‐resolution datasets (XRF‐CS elements and L* lightness values) were smoothed using a Gaussian center‐weighted smoothing function (bandwidth equivalent to 1 cm) prior to 1‐cm interpolation for statistical analysis. Gap‐filling by non‐linear iterative partial least squares PCA (Wold, 1966) was performed to estimate one missing value each for LOI and AFBD, implemented in the R package *pcaMethods* version 1.82.0 (Stacklies et al., 2007).

**TABLE 1:** Proxy datasets from core HM15, their environmental significance and data handling.

Proxy group	Proxy	Environmental significance	Units	Transformation	Standardization
Peat properties	Peat organic matter content by Loss‐on‐ignition (LOI)	Balance of in situ organic production to exogenous inorganic input	Weight percent	None	Yes
	Ash‐free bulk density (AFBD)	Varying degree of peat decomposition	g·cm^−3^	None	Yes
	Optical lightness	Associated to peat humification (decomposition)	L* (0–100)	None	Yes
Anthropogenic proxies	Magnetic susceptibility (Κ)	Balance of diamagnetic organic matter to exogenous iron‐ and nickel‐bearing minerals, esp. airborne pollution	Proportionless SI units	None	Yes
	Pb	Heavy metal contamination from industry and fossil fuel combustion	XRF‐CS element count normalized to inc/coh ratio	log_10_	Yes
	Cu	Heavy metal contamination from industry, esp. regional copper smelting	XRF‐CS element count normalized to inc/coh ratio	log_10_	Yes
	Ti	Dust contamination from land conversion and industry	XRF‐CS element count normalized to inc/coh ratio	log_10_	Yes
	Microcharcoal < 100 μm	Wood combustion products from residential and industrial fires, local to regional sources	Counts	log_10_	Yes
	Microcharcoal > 100 μm	Wood combustion products from residential and industrial fires, inferred locally derived	Counts	log_10_	Yes
	Spheroidal carbonaceous particles	Fossil fuel combustion products	Counts	log_10_	Yes
Pollen	Pollen taxonomic assemblages	Vegetation composition changes	Percent	Hellinger	No
NPPs	Non‐pollen palynomorph assemblages	Fungal, algal, and other biotic tracers of local environmental conditions, e.g., water table depth, and nutrient status	Percent	Cube root	No

Pollen and NPP diagrams were plotted in the R package *rioja* version 0.9–26 (Juggins, 2020). Statistically significant pollen zones were determined by CONISS (stratigraphically constrained cluster analysis by the method of incremental sum of squares; Grimm, 1987). NPPs were allocated to ecological groupings according to previous literature. The richness and diversity of the total pollen assemblage were calculated as the sample‐based rarefaction (Birks & Line, 1992) standardized to a total pollen count of 900 grains (E(T900)) and 10 grains (E(T10)), respectively, following Matthias et al. (2015). The ratio of (E(T900)) and (E(T10)) was then calculated to provide a measure of evenness (Matthias et al., 2015). Rarefaction was implemented in the R package *vegan* version 2.5–7 (Oksanen et al., 2020).

To examine the structure of the multiproxy data and extract the main gradients of change, we use principal component analysis (PCA), a linear method of unconstrained ordination, with attention to suitable data transformations (Juggins & Telford, 2012; Table 1). We consider four groups of proxies, namely, (1) peat properties: LOI, AFBD, L*; (2) anthropogenic proxies: Κ, normalized element counts (Pb, Cu, and Ti), small microcharcoal (<100 μm), large microcharcoal (100–500 μm), SCPs; (3) vegetation, i.e., pollen; and (4) NPPs (Table 1). XRF‐CS counts, charcoal and SCP counts were log‐transformed to improve symmetry, adding 0.5 * lowest value to the normalized XRF‐CS data and 1 to count data (charcoal and SCPs) to avoid zero values. All datasets in groups (1) and (2) were standardized due to measurement in different units. For the pollen data (group 3), pollen of trees and large shrubs as well as rare taxa below a threshold of 1% were excluded so as to focus on the main peatland vegetation components (dwarf shrubs, herbs, mosses, and ferns). The Hellinger transformation was applied to improve suitability for ordination by linear methods (Legendre & Gallagher, 2001). NPP abundances (group 4), which we treat as environmental variables, were cube‐root–transformed to reduce the asymmetry of distributions typical for biological count data with many zeroes and some large values (Juggins & Telford, 2012).

To quantify the main gradients of change in the peat characteristics and anthropogenic proxies, PCA was performed on the proxy groups 1 and 2, and the sample scores on the first principal components (PC1_peat_ and PC1_anthro_, respectively) were extracted. To examine the relationship between the peatland vegetation record and other proxies, we performed PCA on the Hellinger‐transformed pollen data and applied passive fitting of other variables onto the pollen PCA, implemented in the R package *vegan* version 2.5–7 (Oksanen et al., 2020).

Subsequently, constrained ordination using canonical redundancy analysis (RDA) was performed to test the influence of explanatory variables representing local disturbance, climate changes, and industrial anthropogenic drivers on the bog vegetation. The response matrix is the Hellinger‐transformed non‐arboreal pollen data, and the total set of explanatory variables includes local NPP disturbance indicators for (1) grazing and (2) fire; independent climate data for (3) the North Atlantic Oscillation (NAO) and (4) global temperature; and industrial anthropogenic drivers, specifically (5) the first principal component of the anthropogenic proxies, PC1_anthro_, (6) cumulative nitrogen deposition, (7) urban population growth, and (8) bog area. For the NAO (3), we use a highly resolved speleothem record from NW Scotland, which reflects the dynamics of the NAO (Baker et al., 2015). We multiply the original data series (SUcomp of Baker et al., 2015) by −1 since it is inversely correlated to the NAO, such that positive forcing here is associated with positive past NAO phase, a driver for wetter wintertime conditions over NW Britain. For temperature (4), we construct a composite global temperature series (T_global_) of a multiproxy Northern Hemisphere temperature reconstruction (Moberg et al., 2005) for the interval 1280–1979, and the Hadley Center CRUTEM5 observed near‐surface air temperature anomalies for 1850–2020 (Osborn et al., 2021), averaging the anomalies for the overlapping time interval. The records were smoothed using a center‐weighted smoothing function with a Gaussian kernel with a window size of 151 years and a standard deviation of ~30 years (1/5 of the window size). Total nitrogen deposition estimates were derived from the UK CEH simulations (Bell et al., 2021; Tipping et al., 2017), providing values for time slices at 1800, 1900, 1950, 1970, 1990, and 2010 for the 5‐km grid square covering the Holcroft Moss site. Intervening values were derived by linear interpolation, with pre‐1800 values tapering down to a low, non‐negative starting value of 0.25 kg N ha^−1^ year^−1^. The interpolated values were sequentially summed (6) to generate cumulative nitrogen (cuN) values since the cumulative load may be a more critical driver than annual load for terrestrial ecosystems (De Schrijver et al., 2011; Payne, 2014). Population estimates (7) for the nearby city of Manchester, a proxy for regional consumption and material flows, were derived from multiple sources (Arrowsmith, 1985; Walton, 1987). Values for areal extent (ha) of Holcroft Moss (8) between 1845 and 1978 were derived from Bragg et al. (1984). This data constrains well the progressive loss of bog area due to land conversion and peat cutting. Any loss of area prior to 1845 is not documented, but comparison of historical and geological surface mapping suggests this to be minor; since 1978, the areal extent of Holcroft Moss has been effectively stable.

To seek a parsimonious model without overfitting, RDA was performed with forward selection of explanatory variables (RDA_forward_), following confirmation that the total model including all variables (RDA_global_) was significant (Blanchet et al., 2008). The stopping procedure for forward selection was based on the adjusted *R* ^2^ (Peres‐Neto et al., 2006), sequentially adding the best explanatory variable until either the *R* ^2^‐adj begins to decrease, reaches the *R* ^2^‐adj of RDA_global_, or the improvement is insignificant (permutation *p*‐value >.05). The significance of the RDA models and RDA axes was assessed using permutation tests (*n* = 100,000), and variance inflation factors (VIFs) were checked for intercorrelation between variables.

Finally, the variation accounted for by different groups of drivers (local disturbance, climate, and anthropogenic) was estimated by variation partitioning by partial RDA, a powerful tool for assessing the relative importance of anthropogenic drivers of environmental change (e.g., Hall et al., 1999). All explanatory variables were included since variation partitioning on a forward‐selected subset of variables will underestimate shared variation resulting from collinearity in the variables (Legendre & Legendre, 2012). RDA and variation partitioning were implemented in the R package *vegan* following Borcard et al., 2018. Visualization of the variation partitioning as area‐proportional Euler diagrams was performed using the R package Eulerr (Larsson et al., 2021). All significant results are reported at the 95% confidence level.

## 3 RESULTS

### 3.1 Core stratigraphy and chronology

**FIGURE 2:**
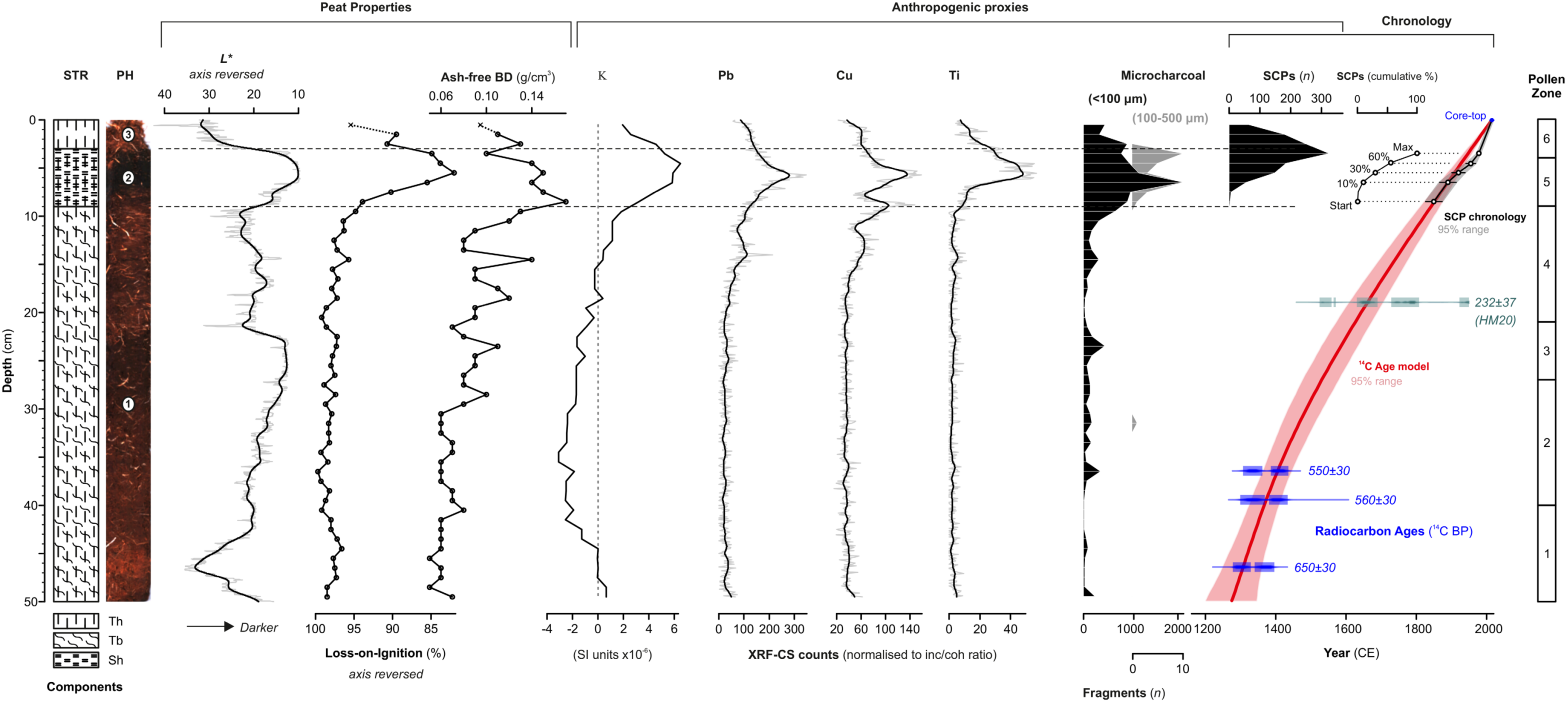
Stratigraphical, geochemical, and age‐model data for Holcroft Moss core HM15, showing, from left to right, the lithostratigraphical core log (STR) with Troels‐Smith components (Th = Turfa herbacea, Tb = Turfa bryophytica, Sh = Substantia humosa), the core photograph (PH) taken by the optical‐line camera on the ITRAX, median brightness (L*) values extracted from the core photograph, organic matter content by loss‐on‐ignition (LOI), ash‐free (organic) bulk density, magnetic susceptibility (Κ), XRF‐CS elemental data for Pb, Cu and Ti normalized to the ratio of incoherent/coherent scattering, microcharcoal concentrations on the pollen slides across two size fractions (<100 μm and 100–500 μm), spheroidal carbonaceous particle counts expressed as counts per sample and cumulative abundance, and age‐depth models based on radiocarbon data (blue radiocarbon ages and red curve) and cumulative SCP abundance (black datapoints and curve). An additional radiocarbon date (green datapoint) from core HM20 recovered at the same location is shown for comparison but not integrated into the age‐depth modeling. Radiocarbon dates show the calibrated age range (2σ line, 1σ boxes); age‐depth models shown with 95% confidence interval (radiocarbon pink, SCP grey). Pollen zones (Figure 3) are plotted for comparison. Dashed horizontal lines delineate the main stratigraphical units, as labeled on the core photograph.

Core HM15 is primarily composed of weakly to moderately humified moss peat, rich in herbaceous plant fragments and *Sphagnum* leaves (Figure 2, Unit 1). Unit 1 is characterized by high organic matter content, variable optical lightness (L*, Figure 2), and gradually increasing AFBD. Heavy metals (Pb and Cu) and dust (Ti) present low values with modest rises toward the top of the unit. The curves for these tracers show similar trajectories with some element‐specific patterns in the timing and abruptness of their rise. Magnetic susceptibility is negative or near zero, reflecting dominance of diamagnetic organic matter (as well as water content of the peat) and virtual absence of ferrimagnetic iron‐ and nickel‐bearing pollutants. Microcharcoal values are low and SCPs absent. Unit 2 reflects a distinctive, dark near‐surface layer comprising well‐humified herbaceous peat without *Sphagnum* leaves. This unit presents minimum organic content and peak AFBD, along with peak values for heavy metals, dust, and combustion proxies (microcharcoal, including large microcharcoal fragments, and SCPs). The uppermost layer (Unit 3) contains moderately humified herbaceous peat and rare *Sphagnum* leaves. Peat properties display a return to higher organic content and lower AFBD, while anthropogenic proxies reveal a decline from peak levels of contaminants including heavy metals, dust, and combustion proxies.

Radiocarbon dating reveals that the core covers the last ca. 700 years of the Common Era (CE) with typical peat accumulation rates of ~0.07 cm year^−1^, equivalent to an average deposition time of ~15 years cm^−1^. We note that the age model agrees well with the newly available date from core HM20, which is not included in the age model (Figure 2). Although this date displays a characteristically complex calibrated age distribution resulting from large fluctuations in the radiocarbon calibration curve in the past few centuries, the age‐depth curve passes through the interval of highest relative probability (1640–1674 CE, 0.481 or 48.1%; Reimer et al., 2020). The SCP chronology (Figure 2) agrees well with the ^14^C chronology and helps refine the age‐model for the upper 10 cm. We splice the chronologies at 6.5 cm depth corresponding to an age of 1890 ± 20 CE and 1890 ± 25 CE on the ^14^C and SCP chronologies, respectively. The 95% confidence interval on age estimates decreases from 130 years at the base of the core to <5 years at the top (Figure 2). To avoid spurious precision, we report all ages rounded to the nearest decade with the 95% confidence interval (rounded to nearest 5 years). As an additional validation of the SCP chronology, we compared the XRF‐CS Pb data to the ^210^Pb dated record of Pb concentration in the Alport Moor peat dome in the Peak District, NW England (Rothwell et al., 2010). A remarkable similarity in the profiles is evident, and the ages of the historical ^210^Pb are in agreement within the uncertainties of the chronologies (see Appendix, Figure S1).

### 3.2 Pollen and NPPs

**FIGURE 3:**
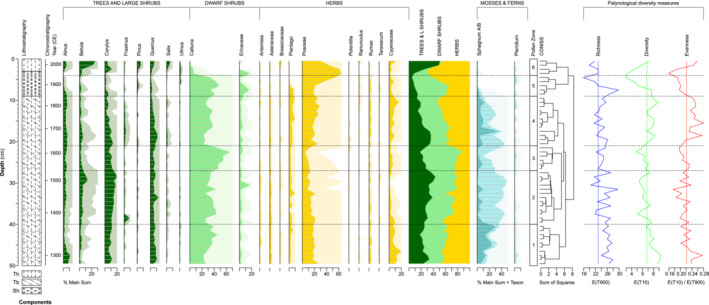
Pollen percentage diagram for core HM15 plotted against depth, grouped according to trees and large shrubs, dwarf shrubs, herbs, and moss and fern spores. Horizontal lines correspond to statistically significant pollen zones. The derived palynological measures of richness, diversity, and evenness are plotted to the right of the diagram.

**FIGURE 4:**
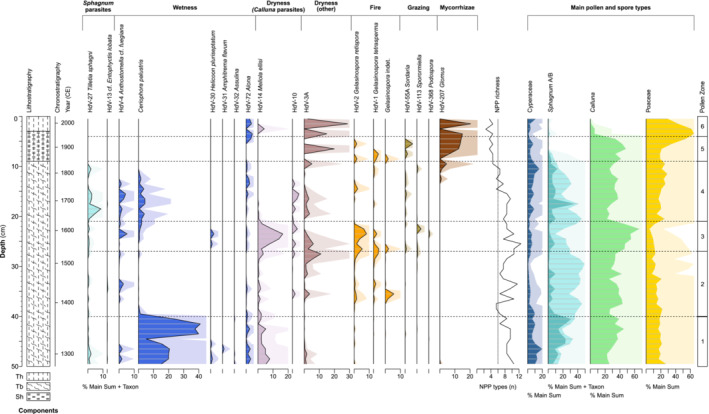
NPP percentage diagram for core HM15 plotted against depth, grouped according to ecological preferences, with NPP richness and main pollen and spore types. Horizontal lines correspond to pollen zones shown in Figure 3.

**FIGURE 5:**
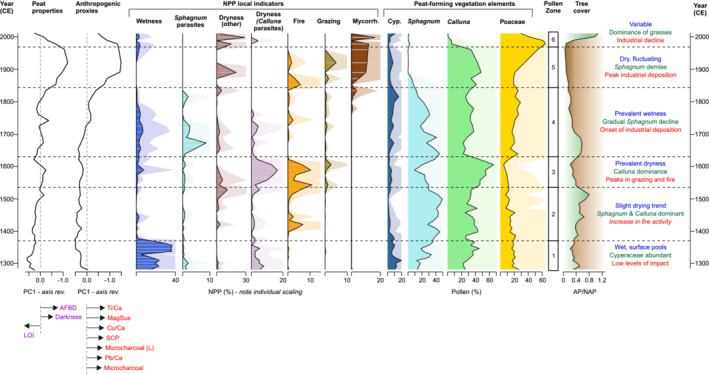
Key environmental variables from core HM15 (PC1 scores for peat properties and anthropogenic deposition proxies; NPPs and pollen) plotted against age; sample ages derived from the ^14^C and SCP chronology (Figure 2). Arrows indicate the PC1 loadings for individual proxies in the peat properties and anthropogenic proxies.

Table 2 summarizes the main changes in pollen, NPPs, charcoal, and geochemical elements. Six zones have been identified according to statistically significant pollen changes (Figure 3). The record is mainly dominated by peat forming elements, namely, *Sphagnum*, *Calluna*, and Cyperaceae along with Poaceae, which shift in abundance across the zones, along with diverse herbaceous and arboreal pollen types reflecting bog, field and woodland ecotypes. A characteristic suite of ombrotrophic bog NPPs has been identified (Figure 4, Table 3), which also shows notable down‐core changes. The pollen and NPP record and inferred environmental changes are described in detail in Section 4.1, making reference to key proxies plotted against age using the HM15 chronology (Figure 5).

**TABLE 2:** Summary of pollen, non‐pollen palynomorphs, charcoal, and geochemical results by pollen zone.

Zone	Depth (cm)	Number of samples	Year (CE)	Main pollen components	Fungi spores and other NPP	Abiotic proxies
1	50–40	10	1280 ± 65–1370 ± 40	Dominance of Cyperaceae, Ericaceae, grasses, and forbs (Poaceae, Asteroideae, and *Rumex*). Some mixed forest is represented by *Alnus*, *Corylus* and *Quercus*. This zone presents the highest values of Cyperaceae and *Sphagnum* A. Richness, diversity and evenness decrease	High abundances of wet indicators (especially *Ceriophora palustris*) and *Sphagnum* parasite fungi (HdV‐27 *Tilletia sphagni*). Also with dryness indicators HdV‐14 *Meliola ellisi* and HdV‐3A	Small percentages of Charcoal type I (<100 μm). Magnetic susceptibility values below zero and the LOI values oscillate. No detectable Pb or Cu values
2	40–27	13	1370 ± 40–1540 ± 50	Rise of dwarf shrubs (Ericaceae) and trees and large shrubs (*Corylus*, *Betula*, and *Alnus*). Decrease of grasses and forbs (Poaceae, Asteroideae). Cyperaceae decrease *Sphagnum* B rises and replaces *Sphagnum* A. Richness strongly fluctuates while diversity and evenness decrease by 33 cm	Decrease in wetness indicators (especially *Ceriophora palustris*). Modest rise in fire indicators (HdV‐1 and HdV‐2 *Gelasinospora* types) and dryness indicators (HdV‐10 and HdV‐3A)	Magnetic susceptibility is around −2 and LOI rises. Pb and Cu values remain at low levels
3	27–21	6	1540 ± 50–1630 ± 45	Grasses (Poaceae) and trees and shrubs decrease (*Betula* and *Corylus*), while dwarf shrubs (*Calluna*, Ericaceae) expand. Cyperaceae also increase. *Sphagnum* A, *Pteridium* and Monolete spores increase while *Sphagnum* B decreases. Richness remains stable, while diversity and evenness decrease	Dry indicators abundant especially *Calluna* parasite HdV‐14 *Meliola ellisi*. Frequent occurrence of fire indicators and grazing indicators. Occasional wetness indicators, with *Ceriophora palustris* increasing toward the top of the zone	Charcoal percentages increase and peaks around 23,5 cm. Magnetic susceptibility slightly increase, while LOI remains constant
4	21–9	12	1630 ± 45–1840 ± 25	Trees and large shrubs recover at the start of the zone and then decrease (*Betula*, *Corylus*, and *Quercus*). Dwarf shrubs (*Calluna*) also decrease, while herbs (Poaceae) rise. *Sphagnum* decrease. Richness and diversity remains stable. Evenness rises between 15–18 cm	Increase in *Sphagnum* parasites and wetness indicators (especially HdV‐4 *Anthostomella* cf. *fuegiana*, Ceriophora palustris and HdV‐72 *Alona*). Marked reduction in HdV‐14 *Meliola* ellisi. Episodic occurrence of other dryness and grazing indicators. Start of rise in mycorrhizae (HdV‐207 *Glomus*) near the top of the zone	Pb and Cu rise slightly while LOI remains constant
5	9–4	5	1840 ± 25–1970 ± 5	Trees and large shrubs continue to decrease (*Alnus*, *Betula*, *Corylus*, and *Quercus*) while dwarf shrubs (*Calluna*, Ericaceae) rise. *Sphagnum* notably decreases. Richness, diversity and evenness strongly decrease	Near absence of *Sphagnum* parasites and wetness indicators. Fluctuating levels of dryness indicator HdV‐3A as well as fire and grazing indicators. Abundant mycorrhizae (HdV‐207 *Glomus*)	Highest values of charcoal and magnetic susceptibility. Together with very high levels of Ti, Pb and Cu. SCP reaches its maximum value
6	4–0	4	1970 ± 5–present (core date, 2015)	Trees and large shrubs rise (*Alnus*, *Betula*, *Corylus*, *Pinus*, *Quercus*, and *Salix*) while Ericaceae remain in low proportions and Poaceae decrease. Cyperaceae slightly increase and *Sphagnum* proportions remain low. Diversity and evenness increase by the end of the zone	Wetness indicators rare except HdV‐72 *Alona*. Dryness indicators (HdV‐3a) and mycorrhizae (HdV‐207 *Glomus*) abundant while fire and grazing indicators disappear	Charcoal, magnetic susceptibility and SCP decreases. LOI rises, while Ti, Pb, and Cu return to low levels

**TABLE 3:** Summary of NPP morphotypes and their indicator value.

Morphotype	Taxonomic group	Indicator value	Indicator group (this study)	References
HdV‐1 *Gelasinospora tetrasperma*	Ascomycota	Grows under dry conditions and on charred material	Fire	Van Geel (1972), Van Geel and Aptroot (2006)
HdV‐2 *Gelasinospora retispora*	Ascomycota	Grows under dry conditions and on charred material	Fire	Shumilovskikh et al. (2015), Van Geel (1972), Van Geel and Aptroot (2006)
HdV‐3A	Fungi	Dry conditions	Dryness	Van Geel (1978)
HdV‐4 *Anthostomella* cf. *fuegiana*	Ascomycota	Associated with raised bog vegetation. Sometimes occurs together with *Eriophorum vaginatum*	Wetness	Van Geel and Aptroot (2006)
HdV‐10	Fungi	Dry conditions, humified peat, and associated with roots of Ericales, especially *Calluna*	Dryness (*Calluna* parasites)	Van Geel (1978)
HdV‐13 (*syn*. cf. *Entophyctis lobata*)	Ascomycota	Found inside the water‐cells of *Sphagnum austinii* leaves	*Sphagnum* parasites	Van Geel (1978)
HdV‐14 *Meliola ellisii*,	Ascomycota	Associated with *Calluna vulgaris* in raised bogs. Indicator of dry conditions	Dryness (*Calluna* parasites)	Van Geel (1978), Van Geel and Aptroot, (2006)
HdV‐27 *Tilletia sphagni*	Ascomycota	Parasite of *Sphagnum*. Especially related to *Sphagnum cuspidatum*. Abundances of these fungi suggest wet conditions and the presence of pools. Indicator of humidity	*Sphagnum* parasites	Van Geel (1978)
HdV‐30 *Helicoon pluriseptatum*	Hyphomycete	Present in peat bogs and marshy places	Wetness	Van Geel (1978)
HdV‐31 *Amphitrema flavum*	Protozoa Rhizopoda	Locally moist conditions. Present in wet phases of *Sphagnum* bogs	Wetness	Charman et al. (2000), Van Geel (1978)
HdV‐32 *Assulina*	Protozoa Rhizopoda	Associated with *Sphagnum* peat	Wetness	Van Geel, (1978)
HdV‐55A *Sordaria*	Ascomycota	Coprophilous fungi; indicates the local presence of herbivores	Grazing	Van Geel and Aptroot, (2006)
HdV‐72 *Alona*	Cladocera	Bog surface pools	Wetness	Van Geel (1976), Van Geel et al. (1983), Van Geel and Van der Hammen (1978)
HdV‐113 *Sporormiella*	Ascomycota	Coprophilous fungi; indicates the local presence of herbivores	Grazing	Van Geel and Aptroot (2006)
HdV‐207 *Glomus*	Glomeromycota	Mycorrhizal fungi	Mycorrhizae	Gelorini et al. (2011)
HdV‐368 *Podospora*	Ascomycota	Coprophilous fungi; indicates the local presence of herbivores	Grazing	Van Geel and Aptroot (2006)
*Ceriophora palustris*	Ascomycota	Indicator of wet, open conditions	Wetness	Ellis and Ellis (1997)

### 3.3 Unconstrained and constrained ordinations

**FIGURE 6:**
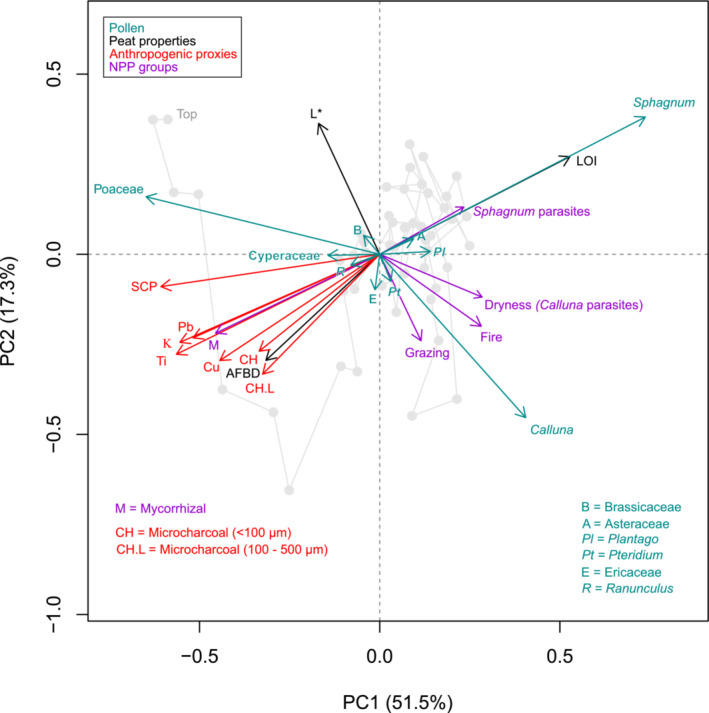
PCA correlation biplot (species scaling) for pollen data with significant environmental fittings (*p* < .05) of peat properties, anthropogenic proxies, and NPP groups.

The first principal components of the peat properties (PC1_peat_) and anthropogenic proxies (PC1_anthro_) capture 64.2% and 74.8% of the variance in their respective proxy groups (Figure 5); no subsequent axes are significant according to a broken‐stick model. PC1_peat_ synthesizes common changes in peat lightness, organic content, and bulk density, thus suggesting a general indication of humification (decomposition) status. PC1_anthro_ reflects the strong common signal of atmospheric deposition of dust, heavy metals, and combustion products. The PCA correlation biplot of the pollen data (Figure 6, Table S1) reveals the main structures in the pollen data. Environmental fitting of the other proxies (Figure 6, Table S2) highlights several significant correlations between the bog vegetation and other proxies. The main bog‐forming taxa of *Sphagnum*, *Calluna*, Poaceae, and to lesser degree Cyperaceae dominate the pollen PCA, which explains 68.8% of the variance across the first two components (PC1 51.5% and PC2 17.3%). There is strong positive loading for *Sphagnum* on PC1, notably correlated with high organic content of the peat (LOI) and high abundance of NPP *Sphagnum* parasites. Poaceae and Cyperaceae show negative loading on PC1 along with anthropogenic proxies for pollution (magnetic susceptibility, Pb, and Cu), dust loading (Ti), and combustion (small and large microcharcoal, SCPs). The abundance of HdV‐207 *Glomus*, a soil mycorrhizal fungal body, is closely allied with the anthropogenic proxies. *Calluna* shows strong negative loading on PC2, aligned with darker peat (lower L*) and NPP indicators for fire, grazing, and dryness indicators (*Calluna* parasites). Overall, negative (positive) scores on PC1 suggest a high (low) degree of anthropogenic influence, while negative (positive) scores on PC2 reflect drier (wetter) conditions associated with greater (lesser) levels of decomposition as well as greater (lesser) local disturbance through fire and grazing. We note that while the wetness and dryness (other) NPP indicator groups do show notable changes through the record (Figure 5), they do not have significant fittings on the pollen PCA.

**FIGURE 7:**
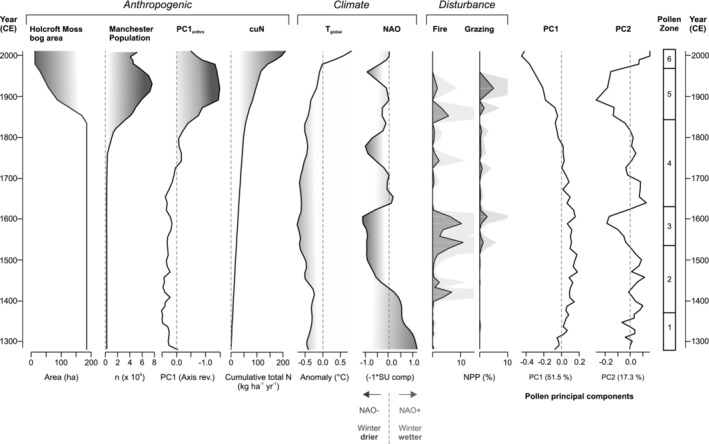
Drivers of environmental change included in the RDA model, accompanied by downcore sample scores on the first and second principal components of the pollen data (cf. Figure 6).

Including all eight environmental variables (Figure 7), RDA_global_ constrains 68.6% of the variance in the pollen dataset. $R_{\mathrm{adj}}^{2}$, accounting for random correlation between variables, is 0.63. Permutation testing confirms that the model and first three RDA axes are significant. All terms are significant, excluding grazing and population. VIFs are low (<10) for fire, grazing, temperature, and NAO but are moderately high (10–20) for PC1_anthro_, cuN, population, and bog area, highlighting correlations between these variables and potential for a more parsimonious model with reduced set of variables. Full model output and significance testing is presented in Table S3. Forward selection of variables (Table S4) yields a similarly powerful model that constrains 65.9% of the variance ($R_{\mathrm{adj}}^{2}$ = 0.62). RDA_forward_ retains five variables, in order of selection: bog area, PC1_anthro_, NAO, cuN, and fire. The model, four axes, and all terms are significant (noting final term Fire, *p* < .1), and VIFs are low (<10; Table S5). As paleoecological datasets may be typically noisy, these statistics indicate that both RDA models perform well and explain a meaningful proportion of the variance in the pollen data. The structure of RDA_global_ and RDA_forward_ is similar apart from the removal of temperature, grazing, and population from the suite of explanatory variables (Figure S2).

**FIGURE 8:**
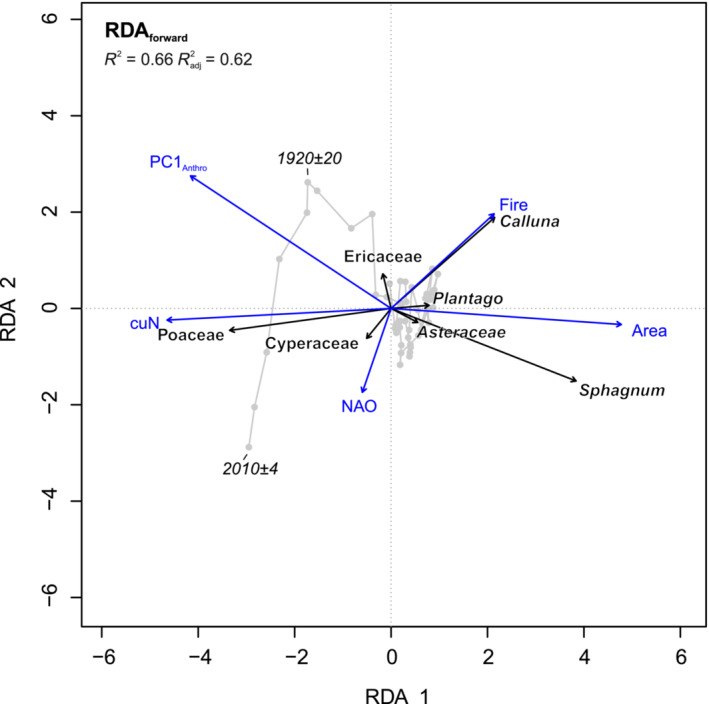
RDA triplot for RDA_forward_; triplot uses species scaling and fitted site scores (linear constraints); black arrows show response variables and blue arrows show explanatory variables.

The first canonical axis (RDA1) of RDA_forward_ explains 48% of the variance (Figure 8) and contrasts higher abundance of *Sphagnum* and *Calluna* with greater bog area (positive scores) vs. higher abundance of Poaceae with increased values of PC1_anthro_ and cuN (negative scores). RDA1 thus relates to anthropic pressures on the bog status. RDA2 explains 11% of the variance and discriminates between higher *Calluna* abundance with greater fire disturbance (positive scores) and higher *Sphagnum* and Cyperaceae abundance with positive NAO (negative scores). RDA2 therefore suggests a hydrological gradient, with wetter conditions associated with positive NAO phases (negative scores) opposed to local fire disturbance under drier conditions (positive scores). RDA3 and RDA4 (not shown) account for 4% and 2% of the variance, respectively. These significant but minor axes explain combined increases in Cyperaceae and *Plantago* in terms of a combination of positive NAO and high bog area (RDA3) and an opposition between *Plantago* and *Pteridium* in terms of a combination of cuN and NAO forcing. The distribution of samples in Figure 8 highlights the important temporal dimension to the RDA results, with a major deviation from positive to negative RDA1 scores from 1810 ± 30 CE.

**FIGURE 9:**
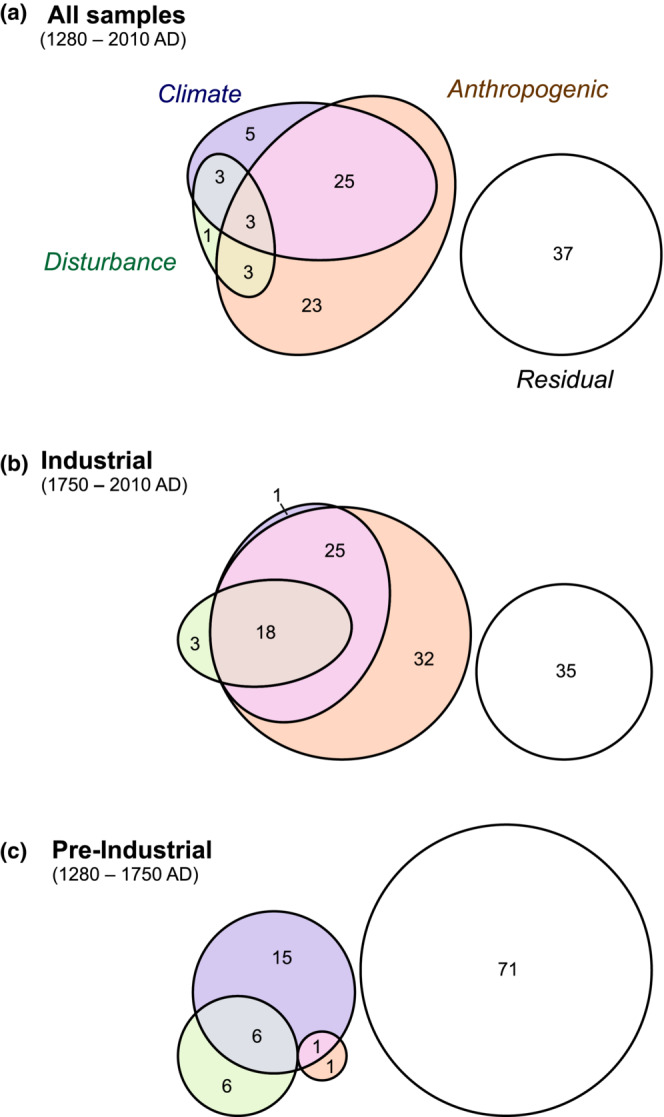
Partitioning of variation across the explanatory variables displayed as area‐proportional Euler diagrams, grouped according to Figure 7. Values in the Euler diagrams are the percentage of variation for each fraction; all fractions reported are significant (*p* < .05).

Partitioning of variation highlights how anthropogenic drivers linked to industry and bog conversion account for the largest fraction of the variation (54% in total, with 31% shared and 23% uniquely), followed by climate drivers (35% in total, with 31% shared and 5% uniquely) and finally disturbance drivers (9% in total, with ~9% shared and <1% uniquely; Figure 9a). A temporal shift in relative importance of the drivers is highlighted by performing discrete analyses on subsets of the record pre‐ and post‐1750 CE (Figure 9b,c). For the pre‐1750 CE samples, climate and disturbance drivers account for larger amounts of variation than the anthropogenic drivers, with 22% attributable to climate (15% uniquely), 12% to disturbance (6% uniquely), and 2% to anthropogenic drivers (1% uniquely; Figure 9c). The total variation explained is low, however, with high residual variation (71%). For the post‐1750 CE samples, anthropogenic drivers account for the largest fraction (66% in total, 32% uniquely), followed by climate (40% in total, 1% uniquely), and then disturbance (7% in total, 3% uniquely), with a smaller residual variation (35%; Figure 9b). In the post‐1750 CE sample set, there is a large overlap between climate and anthropogenic drivers (25%)—associated with the rapid rise of global temperatures in the last century (Figure 7)—and also between all three groups (18%), reflecting significant correlations between the drivers within this interval.

## 4 DISCUSSION

### 4.1 Environmental changes at Holcroft Moss during the last 700 years

#### 4.1.1 Pre‐Industrial conditions

Zone 1 (commencing 1280 ± 65 CE) reflects a landscape dominated by *Calluna*, sphagna, sedges, and grasses with low disturbance (rare NPP fire and grazing indicators; Figure 5). The relatively high abundance of Cyperaceae suggests wet conditions in the peat bog, supported by NPP wetness indicators (HdV‐31 *Amphitrema flavum*, HdV‐32 *Assulina*, and especially *Ceriophora palustris*). Wet conditions are further evidenced in the weakly humified peat condition reflected in minimum scores on PC1_peat_ (Figure 5). The presence of dryness indicators such as HdV‐3A and the *Calluna* parasite HdV‐14 *Meliola ellisi* nevertheless point to local variability in hydrological status. Wet conditions in Zone 1 are consistent with wet phases registered in other peat bogs of northern Britain for this time interval (Barber & Langdon, 2007; McCarroll et al., 2017).

The encroachment of *Calluna* shrubs and diminishment of Cyperaceae point to drier conditions in Zone 2 (from 1370 ± 40 CE) associated with progressively greater humification (rising PC1_peat_, Figure 5). An abrupt reduction in Cyperaceae and NPP wetness indicators and subsequent slight rise in NPP dry indicators supports a shift toward drier conditions at this time. The rise of microcharcoal and *Gelasinospora* spores, which grow under dry conditions and colonize charred material (Van Geel & Aptroot, 2006), points to an increase in fire activity. *Sphagnum* abundance is very high in this zone, associated with a shift in dominant *Sphagnum* spore morphology (Figure 3). This tentative evidence for a shift in dominant *Sphagnum* species at the site is consistent with a wider phenomenon of species replacement across the British Isles, notably the decline of *S*. *austinii*, and should be confirmed by future macrofossil analyses. The decline of *S*. *austinii* occurred generally around the transition to the Little Ice Age (Mauquoy & Barber, 1999; McClymont et al., 2008) but the climatic, anthropogenic, and ecological causes remain contested (Hughes et al., 2008; Swindles et al., 2015).

Zone 3 (from 1540 ± 50 CE) documents a further increase in fire (increased microcharcoal and NPP fire indicators) and grazing activity (Figure 5). Fire and grazing, whether natural or anthropogenic in origin, appear to have favored the dwarf shrub *Calluna*, for which the local presence is confirmed by the peak in the fungal parasite *Meliola ellisii*, typically associated with *Calluna* peat and dry conditions (Van Geel & Aptroot, 2006). The heather‐rich bog vegetation is associated not only with high local disturbance drivers but also a rise in *Pteridium* and reduction in tree pollen (Figure 6). The mid‐16th century CE in Lancashire was marked by population increase (albeit modest and in the context of a sparsely populated region of England), agricultural changes including increased specialization in cattle rearing, and a rapid expansion of woolen and linen trade centered on Manchester (Walton, 1987). The historical context therefore appears favorable for an increase in agropastoral disturbance signals in the paleoecological record. An alternative (or complementary) climatic interpretation can also be considered. NPP dryness indicators and peat properties point to a drier condition leading to high humification, with notable darkening of the peat and rise in AFBD (Figure 2). In general, similarities between the environmental conditions and the reconstructed NAO (Baker et al., 2015) can be observed, with the trend of increasing humification reflected in PC1_peat_ and the rise of *Calluna* associated with the long‐term prevalence of negative NAO phase across the 14th–16th centuries CE (Figure 7). While the NAO is a wintertime rather than growing season phenomenon, wintertime precipitation may nevertheless be an important influence on total P/E ratios which govern peatland hydrology (Charman et al., 2009). As a dominant influence on the position and strength of the North Atlantic westerlies, the NAO has been previously implicated in Holocene peatland hydrological changes (e.g. Roland et al., 2015). In the absence of local precipitation data for this early period, the NAO reconstruction should provide valuable insight into atmospheric circulation variability impacting on the regional climate of NW England.

At the base of Zone 4 (1630 ± 45 CE), a decline of *Calluna* and re‐expansion of *Sphagnum* are documented in a core section with weakly humified peat (low PC1_peat_ scores) and low fire indicators (Figure 5). Values of the fungal wetness indicator *Ceriophora palustris* increased for the first time since their decline in the late 14th century CE (end Zone 1). This probably points to local increase in Cyperaceae since this fungus is commonly associated with sedges (e.g., Barthelmes et al., 2012), and this NPP shows affinities with the pollen curve for Cyperaceae in the record. Values for the *Sphagnum* parasite HdV‐27 *Tilletia sphagni* also reach a maximum by the middle of Zone 4. The transition from *Calluna*‐rich to *Sphagnum*‐rich bog points to a wetter hydrological status at Holcroft Moss, noting that the *Sphagnum* spore record will conflate hummock and pool species, with a loss of ecological precision. Nevertheless, a wet shift at this time is synchronous within age model uncertainties to a marked wet‐shift in bog surface wetness at 1650 CE documented in Northern Ireland and the Cairngorms in Scotland (Barber et al., 2000). These parallels support a common response to climatic forcing, consistent with a shift to prevailing positive NAO as recorded in the Scottish speleothems (Baker et al., 2015; Figure 7). Indeed, similarities between strengthening of the NAO and increase in wetness indicators and decrease in fire indicators extending to present day suggest a persistent influence of Atlantic westerly circulation on bog hydrology and fire activity; this influence is captured by the RDA2 axis (Figure 8). The decrease of fire and grazing indicators along with an increase in tree cover (Figure 6) suggest a reduction in human impact on the site, possibly conditioned by the wetter bog status. Conceivably, catastrophic subsistence crises and epidemics of the early 17th century CE (Walton, 1987) as well as the upheaval of the Civil War (1642–1651) may also be reflected in a reduction in local disturbance indicators and rise in surrounding tree cover (Figure 5).

#### 4.1.2 The industrial revolution

From the mid‐18th century CE onward, vegetation changes within Zone 4 reflect the burgeoning industrial activity of NW England. From 1740 ± 40 CE, PC1_anthro_ scores become positive (Figure 6), reflecting especially the rise of heavy metal concentrations (Pb and Cu) associated with atmospheric deposition from regional coal combustion and smelting activities. The distinctive, early peak in Cu at Holcroft Moss (Figure 2) may be attributed to the site's proximity to early copper smelting centers in nearby St Helens and Warrington (Fletcher & Ryan, 2018). The gradual rise of Poaceae and ruderal taxa (*Plantago*) points to a more disturbed habitat and agricultural activities in the vicinity. A steady decline in tree and large shrub taxa (*Quercus*, *Fraxinus*, and *Salix*) reflects a loss of woodland in the surrounding areas (Figures 3 and 5). The pollen assemblage reveals an increase in diversity and evenness (Figure 3), which may be attributed to an increase in vegetation patchiness and greater number of biotopes in a more fragmented landscape (Matthias et al., 2015). This is associated in this setting with large‐scale drainage and reclamation works pursued by large landowners in the mid‐18th century CE (Birks, 1965). *Sphagnum* declined steadily from the mid‐18th century CE onward, accompanied by a reduction in NPP richness (Figure 4). This loss of diversity may reflect a sensitivity to airborne pollution of some organisms within the peatland microbial and fungal communities, consistent with evidence, for example, from tecamoebae exposure to Pb (Nguyen‐Viet et al., 2008).

By 1840 ± 25 CE (onset Zone 5) perturbation of the bog vegetation increased with a transition from *Sphagnum*‐rich vegetation to vascular plants, initially *Calluna* and subsequently Poaceae. *Sphagnum* spores disappear from the record by 1890 ± 25 CE (corresponding to an absence of *Sphagnum* leaves in stratigraphic Unit 2) in association with high levels of heavy metals and abundant microcharcoal. NPP fire, and grazing indicators point to quite high levels of disturbance on‐site. However, they do not show such a dramatic increase as microcharcoal and large microcharcoal, suggesting that these latter combustion tracers represent a signal of off‐site and regional activity, as reflected in their correlation with other industrial tracers (Figure 6). The lithological shift toward dark substantia humosa and minimum organic content is associated with a slower peat accumulation (Figure 2). The highest PC1_peat_ scores reflect a significant change in peat composition that was heavily influenced by physical deposition of metals, dust, fly ash, and charcoal and possibly by reduced biological productivity. Peak values of PC1_anthro_ (Figure 5) are contemporary with the outstanding rise of the cotton textile industry in the Greater Manchester region as well as population growth and urbanization on a massive scale (Walton, 1987). Atmospheric pollutants (Pb and Cu) reach their maximum in the HM15 record at 1920 ± 25 CE (Figure 2), consistent with the apogee of British cotton textile production in 1913 CE (Sandberg, 1974). In contrast to the progressive rise in Pb and Cu, dust deposition (Ti) rose very sharply at the end of the 19th century CE. Within chronological uncertainties of the age model, this dust increase may reflect activities such as the construction of the Wigan Junction Railway directly through Holcroft Moss, as well as the remarkable construction of the nearby Manchester Ship Canal—a six‐year project that necessitated excavations of 41 M m^3^ undertaken by a labor force of more than 12,000 workers and an arsenal of steam‐powered machinery (Oldham, 1894). Coal smoke might have prompted losses in vegetation, especially *Sphagnum* mosses, and probably hindered the growth of other species such as trees and shrubs. Indeed, contemporary accounts note “*fruitful vales where vegetation flourished … have been changed by the deleterious compounds of coal‐smoke into barren deserts*” (Emrys‐Jones, 1882: 3), in (Mosley, 2008). A marked reduction in palynological richness, evenness, and diversity is documented within Zone 5, suggesting a decline in vegetal biodiversity. Although many caveats exist for the interpretation of palynological diversity measures, including plant taxonomic differences and differential pollen representation (Felde et al., 2016), this pattern may be suggestive of both a reduction in α‐diversity associated with dominance of vascular plants on the raised bog and in β‐diversity in response to the cumulative impacts of land reclamation, industrialization, and urbanization in the wider landscape.

The 20th century CE at Holcroft Moss was marked by the steady rise of Poaceae (Figure 5). The progressive colonization of bogs and heaths by grasses is a widespread phenomenon in the British Isles, particularly associated with invasion by *Molinia caerulea*, which is currently abundant at Holcroft Moss. The invasive capability of *Molinia* was already discussed in the early 20th century CE in northern England (Jefferies, 1915). In blanket bogs of southern and south‐western Wales, *Molinia* dominance was reached within the 20th century CE, pointing to novel drivers of change not evidenced in earlier times (Chambers et al., 2007, 2013). Similar findings have been made in the South Pennines, where pollen records evidence a regime shift in moorland vegetation with replacement of *Calluna* by grasses (Davies, 2016). In the case of upland heath on Exmoor, paleoecological research reveals that *Molinia* expansion was not a uniquely recent phenomenon but part of a fluctuating alternation of *Molinia* and *Calluna* dominance that preceded a rise to dominance in the 20th century CE (Chambers et al., 1999). Although we cannot confirm the species in the absence of macrofossil data, the recent dominance of grasses at Holcroft Moss appears unprecedented in the framework of the last 700 years, although some spikes in grass pollen were observed in the undated deeper biostratigraphy of Holcroft Moss (Birks, 1965). In common with the blanket bogs in Wales, the rise of grasses at Holcroft Moss above the longer‐term baseline was essentially a 20th century CE phenomenon, occurring in parallel with loss of intact peat bog to agriculture, drainage, and peat cutting (Bragg et al., 1984). Coprophilous and fire‐related spores and microcharcoal suggest that local disturbance was significant during this period, although these cannot account for the vegetation composition since elevated levels of these factors did not favor a similar expansion of grasses in the late 16th to early 17th centuries CE, when these disturbance factors promoted instead a heather‐dominated vegetation (Zone 3). However, industrial atmospheric deposition was much higher in Zone 5 and might have favored the competitive dominance of Poaceae over other NAP taxa associated with human activity (see also Section 4.2). The rise to dominance of Poaceae is accompanied by a remarkable increase in arbuscular mycorrhizal fungi (HdV‐207 *Glomus*). While considered an erosion indicator in lake sediments, HdV‐207 *Glomus* in ombrotrophic bogs such as Holcroft Moss may derive from fungi that colonized the root structures of plants growing on the bog surface and thus may penetrate down into older peat (Kołaczek et al., 2013).

The uppermost Zone 6, extending from 1970 ± 5 CE to present, documents the decline of *Calluna* and other Ericaceous plants and presents the peak abundance of Poaceae. NPP fire, and grazing indicators are low, pointing to reduced local disturbance at the site compared with earlier periods. We do not detect a signal in coprophilous fungi of the recent stocking of Holcroft Moss with sheep, although chronologically this might only be expected in the single topmost sample. SCPs peak in the regionally characteristic pattern near the base of Zone 6, discriminating clearly between the historical maximum of heavy metals, charcoal, and dust deposition in the final 19th to early 20th centuries CE and the mid‐to‐late 20th century CE maximum of high‐temperature (>1000 °C) combustion of coal and oil, characteristic of energy production and heavy industry (Swindles et al., 2015). Overall, however, the substantial decline of PC1_anthro_ scores (Figure 5) reflects the decline of regional industry and implementation of air pollution control measures (Douglas et al., 2002). Another important perturbation near Holcroft Moss was the construction of the M62 motorway in 1971. The slight rise of *Sphagnum* around that time suggests a counter‐intuitive slight recovery of moss‐favoring conditions, which according to Valentine et al. (2013) could be related with wetter conditions resulting from the creation of the bund which separates the bog from the motorway. The substantial rise of tree and large shrub pollen (Figure 5), notably *Betula*, *Pinus*, and *Salix*, is also a likely reflection of the diminished size of Holcroft Moss, with arboreal planting on the bund and establishment of the present‐day boundary woodlands (Figure 1). These same factors may also underline an increase in the richness of local biotopes reflected in increased palynological richness, diversity, and evenness (Figure 3).

### 4.2 Drivers of vegetation change

The HM15 record not only documents environmental changes in a key site of restoration interest in the Manchester Mosses lowland bogs but also provides a test‐bed for exploring the role of different drivers of change across the pre‐Industrial to post‐Industrial eras. Some key points emerge from the statistical analysis, specifically:

Hydrological (NAO) forcing and local disturbance factors, influential on RDA2 (Figure 8), predominate in the pre‐Industrial record with drier conditions favoring *Calluna* development. Corresponding darkening of the peat reflects greater humification, a characteristic association linked primarily to the ecological preferences of *Calluna* but also the influence of woody peat‐formers on decomposition rates (Yeloff & Mauquoy, 2006). Drier phases appear to have been more favorable for local fire activity, consistent with a greater prevalence of fire due to reduced fuel moisture content (Davies & Legg, 2011). The association also suggests a positive ecological response of *Calluna* to fire activity in this setting, perhaps due to resprouting from meristems or enhanced seed germination (Whittaker & Gimingham, 1962). This insight is valuable since the implications of burning for *Calluna* regeneration are complex and contested (Davies, 2016), having also been implicated more widely in the demise of heather moorland across northern Britain (Stevenson & Rhodes, 2000). Neither hydrological changes nor fire activity are necessarily driven by climate alone, and within the wider historical context, coherent economic and population changes can be invoked to explain the changing incidence of local disturbance, including fire‐lighting and grazing. However, the good correspondence between weakening of the NAO and enhanced local fire activity supports a primacy of atmospheric climate forcing on decadal and longer timescales. The association of dry conditions with grazing indicators may also suggest that drier conditions offered more favorable opportunities for exploitation of Holcroft Moss, such as better access for cattle grazing.

From the late 18th to early 19th centuries CE onward, anthropogenic forcing associated with industrial activity and land use change dominates, reflected by a definitive shift from positive to negative scores on RDA1 (Figure 8) and dominance of these drivers in the partitioning of variation (Figure 9b). This transition furthermore reflects a shift from predominant local to regional forcing factors, as well as a shift from episodic or fluctuating disturbances to progressive, sustained anthropogenic impacts linked to the industrialization of the region (Figure 9b,c). As such, we do not exclude a role for anthropogenic activity prior to the Industrial era but rather highlight a key transition to a human‐dominated landscape with the ascendancy of industrial over agropastoral impacts by the early 19th century CE. This characterization suggests that the Holcroft Moss ecosystem may usefully be viewed as a socio‐ecological system with a historical trajectory that was strongly influenced by changes in human activity and behavior, and a shift from “pulse” to “press” drivers (Collins et al., 2011). This trajectory reflects the progressive incorporation of insular rural areas within the growing sphere of industrial influence, as the Manchester region transformed into the hub of the Industrial Revolution (Walton, 1987). In the global context, this environmental transition was notably early (Snowball et al., 2014), in common with the Lower Swansea Valley in South Wales, another center of early industrialization (Rosen & Dumayne‐Peaty, 2001).

The key biological signal of regional industrialization was the decline of *Sphagnum*. *Sphagnum* abundance in the record is directly opposed to the input of atmospheric deposition proxies, especially Ti, Pb, and magnetic susceptibility (Figure 6). *Sphagnum* decline appears as the earliest and most sensitive vegetation response to industrial deposition, notable already from the mid‐18th century CE. Due to the strong correlations between the anthropogenic proxies (Figure 6), it is not possible to identify a unique driver for the *Sphagnum* decline, but several cumulative factors may be implicated. Heavy metals accumulate strongly in bryophytes as they possess no cuticle to moderate the uptake of heavy metals and derive water via direct absorption by the whole plant body (Tyler, 1990). Injurious effects of heavy metals include ultrastructural cellular impacts, metabolic changes, and increased reactive oxygen species, leading to physiological and oxidative stress (Stanković et al., 2018). Importantly, the high levels of heavy metals documented, exceeding 1100 ppm and 450 ppm for Pb and Cu, respectively, (Fletcher & Ryan, 2018) were almost certainly accompanied by significant sulfur emissions and acid deposition of sulfur solution products, which also strongly inhibit *Sphagnum* growth (Ferguson et al., 1978). Low tolerance to dust and nitrogen loading (Farmer, 1993; Hughes et al., 2008; Zhou et al., 2021) may also be factors in the decline at Holcroft Moss. Ultimately, the primacy of different factors may be difficult to assess and may be a moot point in the historical perspective due to the broadly collinear rise in anthropogenic proxies (Figure 2), such that synergistic effects of heavy metal, dust, and acid deposition can be inferred (Raeymaekers & Glime, 1987). The findings from the lowland area of Manchester form part of the wider pattern of dramatic decline of *Sphagnum* across upland bogs of the Pennines in response to a common forcing, specifically atmospheric pollution emanating from the industrial centres of NW England (Tallis, 1964, 1987).

Two factors emerge as strong predictors for the rise in Poaceae at Holcroft Moss: cumulative nitrogen loading (cuN) and loss of bog area, both strongly influential on RDA1 (Figure 8). The significance of bog area in the RDA models (indeed primacy in forward selection of variables) furthermore suggests a likely suite of inter‐related problems that affected the functional integrity of Holcroft Moss, including impaired hydrological status and reduced biological connectivity. Drying of the bog in conjunction with nitrogen enrichment is likely to have stimulated the growth of vascular plants and increased the competitive dominance of grasses at the site (Tomassen et al., 2003; Wooliver et al., 2016). Looking forward, the rising temperatures of ongoing climate change and local perturbations are expected to enhance the dominance of vascular plants at the expense of bryophytes (Leroy et al., 2019). While temperature was not retained in RDA_forward_, the large shared partition for climate and anthropogenic forcing in the variation partitioning highlights a substantial overlap between these drivers after 1750 CE (Figure 9b). As such, grass dominance may reflect powerful feedbacks in the bog system between impaired hydrological status, enhanced nitrogen availability, and rising temperatures. Our findings echo inferences of 20th century CE regime shift toward dominance of competitive grass taxa in bogs of the Peak District (Davies, 2016).

### 4.3 Conservation implications

This study reveals that the present‐day vegetation composition at Holcroft Moss remains different from pre‐Industrial times despite the reduction in industrial deposition and thus supports the need for restoration actions. Constrained ordination with forward selection helps identify the most powerful drivers of change and thus may help establish priorities for restoration and conservation for Holcroft Moss and the Manchester Mosses (McCarroll et al., 2016). The primacy of bog area in RDA_forward_ highlights the injurious effects of historical bog conversion at the site and underscores the need to increase the area of the natural reserves, both to restore hydrological function and improve connectivity between reserves (Figure 1). Reversing the impacts of fragmentation should be a priority to increase the resilience of existing habitat patches and species' populations (Sutherland et al., 2010). The importance of cuN is highly problematic due to the difficulty for control or mitigation. In particular, the immediate proximity to a major motorway represents an intractable problem, although increase in electric vehicles may help reduce the burden on the site in coming decades (Mehlig et al., 2021). Nevertheless, modeling studies suggest that nitrogen deposition will continue to drive vegetation changes in coming years (Stevens et al., 2016). In contrast, the atmospheric deposition of heavy metals, dust, and combustion products (PC1_anthro_) has substantially diminished since the mid‐20th century C. The diminishing burden of these products on the site therefore represents a positive context for restoration actions. However, open questions remain about the legacy of highly polluted sub‐surface peat layers on below ground microbial communities (Elliott et al., 2015).

In terms of desirable vegetation composition, a focus on *Sphagnum* restoration is justified in redressing a major biological legacy of the Industrial Revolution at this site. Conservation measures to improve site hydrology and raise water tables are important for the recolonization of peat‐forming species such as *Sphagnum* (Holden et al., 2004); indeed, progress has been made at Holcroft Moss in the restoration of *Sphagnum*, and the paleoecological record reflects a modest increase in *Sphagnum* in the uppermost peat layers. However, *Sphagnum* recovery may be hindered by various factors, such as ongoing deposition of atmospheric nitrogen (Adams & Preston, 1992; Caporn et al., 2006), sub‐surface pollution, and small bog area, such that alternative restoration targets might be necessary. A noteworthy feature of the 700 years pollen record is the prevailing high abundance of *Calluna* which contrasts with its rarity on the site today. The tolerance of drier conditions as well as local disturbance through fire and grazing make it a candidate for restoration activities, especially as the IPCC (2021) projects an increase in precipitation variability and extremes, along with drier summers and increased temperatures.

Our study relies on pollen as the primary tracer of past vegetation and NPPs for their window into a diverse group of biota. However, future studies may incorporate other proxies to refine the understanding of changes in the bog ecosystem, including testate amebae to improve the knowledge of water table changes and plant macrofossils to reconstruct vegetation changes with greater taxonomic resolution. Sedimentary DNA would also allow a higher taxonomic resolution than pollen, and it would be particularly interesting to use universal eukaryotic markers to reveal the hidden diversity of peat bogs (Garcés‐Pastor et al., 2019). By characterizing the group of organisms that coexist in the diverse peat bog microhabitats (fungi, diatoms, arthropods, and other microeukaryotes that live on the bryophytes), we can better understand how those ecosystems responded to human impact across the Industrial Revolution.

## 5 CONCLUSIONS

The study of biotic and abiotic proxies in core HM15 reveals the peat bog response to disturbance, climate, and anthropogenic drivers across the trajectory of industrialization in NW England. Before the Industrial Revolution, the bog vegetation responded to changes in prevailing NAO phase and fire activity that alternately favored *Sphagnum* and *Calluna*. From the mid‐18th century CE onward, sustained rise in anthropogenic impacts including atmospheric deposition of dust, heavy metals, and nitrogen, and loss of bog area to land conversion provoked two major vegetation changes: first, the decline of *Sphagnum* in the 19th century CE and subsequently the rise of grasses in the 20th century CE. Collectively, anthropogenic drivers linked to industry and peatland conversion account for the largest fraction of variation in the vegetation data, revealing the dominant imprint of the regional transformation of NW England on the lowland raised bog environment. Although the levels of many industrial tracers in the peat have decreased strongly, we do not observe a return to pre‐Industrial vegetation composition, notably with still reduced levels of both *Sphagnum* and *Calluna*. Restoration actions are needed to improve site hydrology and diversity, consistent with activities underway at the site. However, external factors such as N deposition and temperature rise in the 21st century CE pose a challenge for restoration and may contribute to unexpected outcomes.

## AUTHOR CONTRIBUTIONS

**Sandra Garcés‐Pastor:** Data curation (equal); formal analysis (equal); investigation (equal); writing – original draft (equal); writing – review and editing (equal). **William Fletcher:** Formal analysis (equal); funding acquisition (equal); investigation (lead); methodology (equal); project administration (equal); supervision (equal); writing – original draft (equal); writing – review and editing (equal). **Peter Ryan:** Formal analysis (equal); funding acquisition (lead); investigation (equal); methodology (equal); project administration (equal); resources (lead); writing – original draft (equal); writing – review and editing (equal).

### OPEN RESEARCH BADGES

This article has earned an Open Data badge for making publicly available the digitally‐shareable data necessary to reproduce the reported results. The data is available at [https://doi.org/10.48420/21890748.v1].

## Supporting information


Figure S1

Figure S2

Tables S1‐S5

## Data Availability

The data that support the findings of this study will be uploaded in the repository of the University of Manchester Library (https://doi.org/10.48420/21890748.v1).
