# Supplementary figures and images for: Ecological impacts of the industrial revolution in a lowland raised peat bog near Manchester, NW England
Source: Ecol Evol. 2023 Feb 14;13(2):e9807. doi: 10.1002/ece3.9807 (PMC9926178; doi:10.1002/ece3.9807)

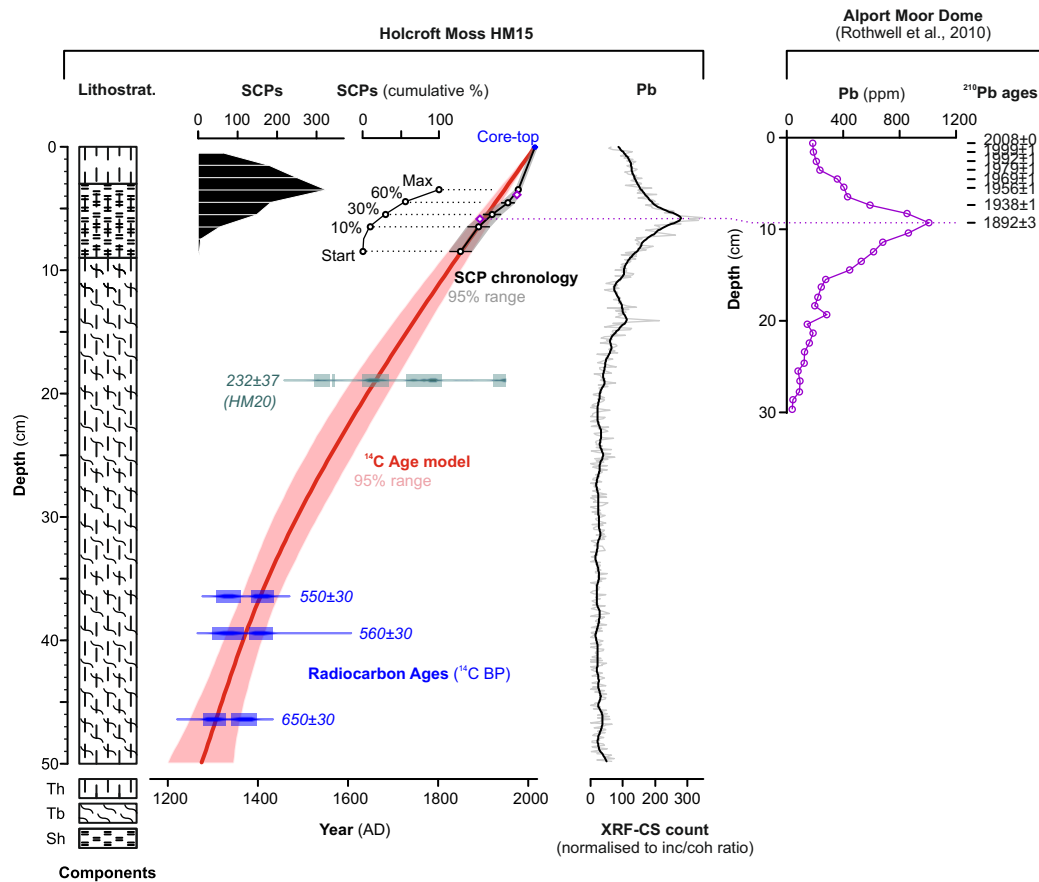

Supplement: Supplementary file 1 — Figure S1 [file ECE3-13-e9807-s002.pdf]

a

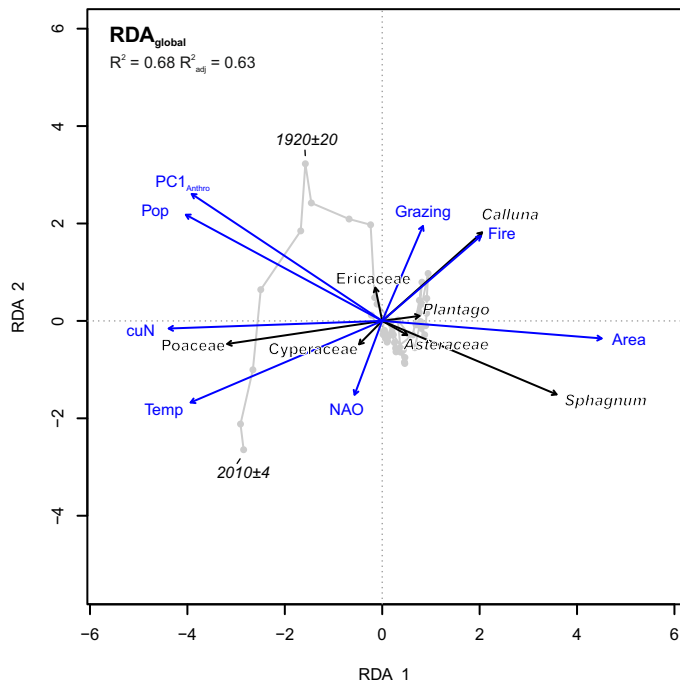

b

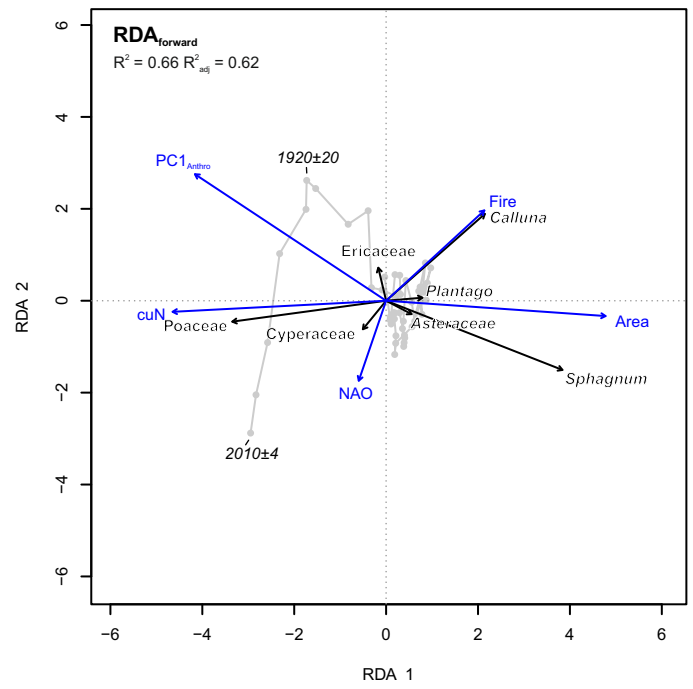

Supplement: Supplementary file 2 — Figure S2 [file ECE3-13-e9807-s003.pdf]
