# Supplementary material for: Ecological impacts of the industrial revolution in a lowland raised peat bog near Manchester, NW England
Source: Ecol Evol. 2023 Feb 14;13(2):e9807. doi: 10.1002/ece3.9807 (PMC9926178; doi:10.1002/ece3.9807)
Supplement: Supplementary file 3 — Tables S1‐S5 [file ECE3-13-e9807-s001.docx]

**Supplementary Table 1**. Results of PCA of pollen data for first six components. PC1 and PC2 are significant according to a broken-stick model of random variance.

| **Partitioning of variance:** | | | | | | |
| --- | --- | --- | --- | --- | --- | --- |
|  | Variance | Proportion |  |  |  |  |
| Total | 0.110 | 1 |  |  |  |  |
| Unconstrained | 0.110 | 1 |  |  |  |  |
|  |  |  |  |  |  |  |
|  | PC1 | PC2 | PC3 | PC4 | PC5 | PC6 |
| **Eigenvalues, and their contribution to the variance:** | | | | | | |
| Eigenvalue | 0.405 | -0.453 | 0.051 | -0.056 | 0.039 | -0.035 |
| Proportion explained | -0.013 | -0.098 | -0.297 | 0.186 | -0.078 | 0.087 |
| Cumulative proportion | 0.009 | -0.027 | -0.017 | 0.044 | -0.014 | -0.003 |
|  |  |  |  |  |  |  |
| ***Species scores:** |  |  |  |  |  |  |
| *Calluna* | 0.405 | -0.453 | 0.051 | -0.056 | 0.039 | -0.035 |
| Ericaceae | -0.013 | -0.098 | -0.297 | 0.186 | -0.078 | 0.087 |
| Amaranthaceae | 0.009 | -0.027 | -0.017 | 0.044 | -0.014 | -0.003 |
| *Artemisia* | 0.041 | 0.042 | 0.034 | -0.050 | 0.015 | 0.075 |
| Asteraceae | 0.094 | 0.043 | 0.069 | 0.016 | 0.003 | 0.021 |
| Brassicaceae | -0.045 | 0.053 | 0.018 | 0.034 | -0.091 | -0.015 |
| Cyperaceae | -0.143 | -0.003 | 0.275 | 0.251 | -0.060 | -0.055 |
| *Helianthemum* | -0.046 | 0.007 | -0.005 | -0.006 | -0.002 | 0.026 |
| *Plantago* | 0.140 | 0.008 | 0.010 | 0.160 | 0.189 | 0.081 |
| Poaceae | -0.649 | 0.160 | 0.004 | -0.066 | 0.069 | 0.023 |
| *Potentilla* | 0.054 | 0.008 | -0.038 | 0.026 | -0.054 | -0.018 |
| *Ranunculus* | -0.080 | -0.030 | 0.001 | 0.086 | 0.018 | 0.013 |
| *Rumex* | 0.011 | -0.012 | 0.073 | 0.041 | 0.073 | 0.026 |
| *Taraxacum* | 0.013 | -0.028 | -0.021 | 0.032 | 0.035 | 0.018 |
| *Sphagnum* | 0.737 | 0.381 | 0.007 | 0.007 | -0.009 | 0.003 |
| *Pteridium* | 0.031 | -0.075 | 0.180 | -0.073 | -0.092 | 0.197 |
| Monolete | -0.035 | -0.018 | 0.018 | 0.023 | -0.015 | 0.013 |
|  | | | | | | |
| ***Site scores (depth in cm), weighted sums of species scores:** | | | | | | |
| 0.5 | -0.588 | 0.374 | -0.014 | 0.476 | -0.544 | -0.362 |
| 1.5 | -0.629 | 0.373 | 0.073 | -0.368 | 0.180 | -0.306 |
| 2.5 | -0.571 | 0.172 | 0.117 | -0.458 | 0.017 | 0.187 |
| 3.5 | -0.503 | 0.166 | -0.741 | -0.058 | -0.172 | 0.195 |
| 4.5 | -0.437 | -0.376 | 0.013 | -0.489 | 0.161 | 0.069 |
| 5.5 | -0.295 | -0.438 | 0.244 | -0.277 | -0.086 | 0.092 |
| 6.5 | -0.251 | -0.655 | -0.320 | 0.108 | 0.129 | -0.246 |
| 7.5 | -0.108 | -0.311 | -0.485 | 0.397 | 0.295 | 0.248 |
| 8.5 | -0.062 | -0.326 | -0.050 | 0.136 | 0.088 | 0.443 |
| 9.5 | -0.101 | -0.102 | -0.083 | 0.411 | 0.406 | -0.148 |
| 10.5 | -0.073 | -0.003 | 0.386 | 0.293 | 0.168 | 0.190 |
| 11.5 | -0.052 | 0.058 | 0.183 | 0.068 | 0.036 | 0.299 |
| 12.5 | 0.029 | 0.026 | -0.026 | 0.158 | 0.325 | -0.321 |
| 13.5 | 0.025 | 0.107 | -0.029 | 0.116 | 0.358 | -0.299 |
| 14.5 | 0.040 | 0.021 | 0.066 | 0.046 | 0.291 | 0.426 |
| 15.5 | 0.006 | -0.088 | -0.053 | 0.215 | 0.087 | -0.387 |
| 16.5 | 0.069 | -0.054 | -0.181 | 0.170 | 0.178 | -0.246 |
| 17.5 | 0.117 | 0.195 | 0.054 | -0.153 | 0.254 | 0.284 |
| 18.5 | 0.019 | 0.187 | 0.068 | 0.231 | 0.268 | -0.338 |
| 19.5 | 0.134 | 0.169 | -0.114 | 0.174 | -0.264 | 0.147 |
| 20.5 | 0.083 | 0.305 | -0.073 | -0.154 | 0.208 | 0.270 |
| 21.5 | 0.188 | -0.036 | -0.172 | -0.079 | -0.254 | -0.072 |
| 22.5 | 0.214 | -0.402 | -0.171 | -0.154 | -0.224 | -0.149 |
| 23.5 | 0.090 | -0.449 | 0.205 | -0.112 | -0.160 | -0.022 |
| 24.5 | 0.164 | -0.239 | 0.020 | -0.109 | -0.045 | -0.102 |
| 25.5 | 0.142 | -0.125 | 0.225 | -0.180 | -0.134 | -0.192 |
| 26.5 | 0.156 | -0.094 | 0.010 | -0.126 | -0.038 | 0.006 |
| 27.5 | 0.133 | 0.010 | -0.029 | 0.022 | -0.169 | -0.030 |
| 28.5 | 0.242 | 0.105 | -0.186 | -0.082 | -0.037 | 0.014 |
| 29.5 | 0.214 | 0.217 | -0.171 | -0.236 | 0.223 | -0.076 |
| 30.5 | 0.186 | 0.160 | -0.568 | 0.110 | -0.386 | 0.225 |
| 31.5 | 0.249 | 0.023 | -0.116 | -0.133 | -0.027 | -0.108 |
| 32.5 | 0.121 | 0.271 | -0.063 | -0.066 | 0.344 | 0.196 |
| 33.5 | 0.179 | 0.129 | -0.071 | -0.184 | -0.062 | -0.149 |
| 34.5 | 0.142 | 0.052 | -0.058 | -0.137 | -0.113 | -0.033 |
| 35.5 | 0.124 | -0.038 | 0.018 | -0.002 | 0.076 | 0.273 |
| 36.5 | 0.127 | 0.039 | -0.110 | -0.285 | -0.483 | -0.132 |
| 37.5 | 0.201 | 0.097 | -0.038 | -0.237 | 0.070 | -0.195 |
| 38.5 | 0.085 | 0.241 | 0.075 | 0.000 | -0.008 | -0.003 |
| 39.5 | 0.068 | 0.181 | 0.058 | 0.183 | -0.068 | 0.128 |
| 40.5 | 0.116 | 0.077 | 0.144 | -0.115 | 0.127 | -0.155 |
| 41.5 | 0.069 | 0.074 | 0.225 | 0.103 | 0.083 | -0.001 |
| 42.5 | 0.046 | -0.161 | 0.135 | -0.011 | -0.150 | -0.101 |
| 43.5 | 0.025 | -0.030 | 0.062 | 0.015 | -0.010 | 0.097 |
| 44.5 | 0.093 | 0.087 | 0.201 | -0.162 | 0.020 | -0.122 |
| 45.5 | 0.036 | 0.089 | 0.243 | -0.019 | -0.122 | -0.106 |
| 46.5 | -0.070 | -0.097 | 0.339 | 0.384 | -0.205 | -0.131 |
| 47.5 | -0.044 | 0.034 | 0.244 | 0.229 | -0.286 | 0.423 |
| 48.5 | -0.037 | 0.004 | 0.331 | 0.223 | -0.198 | 0.122 |
| 49.5 | -0.111 | -0.017 | 0.183 | 0.118 | -0.145 | 0.202 |
| **Scaling 2 for species and site scores. Species are scaled proportional to eigenvalues. Sites are unscaled: weighted dispersion equal on all dimensions. General scaling constant of scores: 1.524* | | | | | | |

**Supplementary Table 2**. Environmental fitting (vectors) of co-registered proxies on the first two axes (PC1, PC2) of the pollen PCA. Results significant at the 95% confidence level are plotted in Fig. 5.

| **Proxy** | **PC1** | **PC2** | **r^2^** | **Pr(>r)** | **Significance** |
| --- | --- | --- | --- | --- | --- |
| L* | -0.423 | 0.906 | 0.347 | 5.00E-05 | *** |
| LOI | 0.890 | 0.457 | 0.761 | 1.00E-05 | *** |
| AFBD | -0.732 | -0.682 | 0.403 | 3.00E-05 | *** |
| MS | -0.915 | -0.403 | 0.794 | 1.00E-05 | *** |
| Microchar | -0.779 | -0.627 | 0.398 | 3.00E-05 | *** |
| Microchar.L | -0.699 | -0.716 | 0.467 | 5.00E-05 | *** |
| SCP | -0.989 | -0.146 | 0.814 | 1.00E-05 | *** |
| Pb | -0.913 | -0.409 | 0.697 | 1.00E-05 | *** |
| Cu | -0.833 | -0.554 | 0.613 | 1.00E-05 | *** |
| Ti | -0.897 | -0.441 | 0.855 | 1.00E-05 | *** |
| *Sphagnum* parasites | 0.875 | 0.484 | 0.161 | 0.015 | * |
| Wetness | 0.969 | 0.248 | 0.011 | 0.770 |  |
| Dryness *(Calluna* parasites) | 0.933 | -0.361 | 0.210 | 0.004 | ** |
| Dryness (other) | -0.991 | 0.132 | 0.039 | 0.399 |  |
| Grazing | 0.448 | -0.894 | 0.153 | 0.020 | * |
| Fire | 0.823 | -0.568 | 0.258 | 0.002 | ** |
| Mycorrhizae | -0.902 | -0.431 | 0.549 | 1.00E-05 | *** |
| Significance codes: 0 ‘***’ 0.001 ‘**’ 0.01 ‘*’ 0.05 ‘.’ 0.1 ‘ ’ 1 | | | | | |

**Supplementary Table 3**. Model output and significance testing for RDA_global_.

| **Call: rda(formula = NAP ~ Fire + Grazing + Temp + NAO + CuN + PC1.Anthro + Pop + Area, data = Env.all)** | | | | | | | |  |  |
| --- | --- | --- | --- | --- | --- | --- | --- | --- | --- |
| **Partitioning of variance:** | | |  |  |  |  |  |  |  |
|  | Variance | Proportion |  |  |  |  |  |  |  |
| Total | 0.110 | 1.000 |  |  |  |  |  |  |  |
| Constrained | 0.076 | 0.686 |  |  |  |  |  |  |  |
| Unconstrained | 0.035 | 0.314 |  |  |  |  |  |  |  |
|  |  |  |  |  |  |  |  |  |  |
| **Eigenvalues and their contribution to the variance:** | | | | |  |  |  |  |  |
|  | RDA1 | RDA2 | RDA3 | RDA4 | RDA5 | RDA6 | RDA7 | RDA8 |  |
| Eigenvalue | 0.054 | 0.013 | 0.005 | 0.002 | 0.001 | 0.001 | 0.000 | 0.000 |  |
| Proportion explained | 0.487 | 0.117 | 0.042 | 0.023 | 0.008 | 0.005 | 0.003 | 0.001 |  |
| Cumulative proportion | 0.487 | 0.604 | 0.646 | 0.668 | 0.676 | 0.681 | 0.685 | 0.686 |  |
|  |  |  |  |  |  |  |  |  |  |
|  | PC1 | PC2 | PC3 | PC4 | PC5 | PC6 | PC7 | PC8 | PC9 |
| Eigenvalue | 0.010 | 0.007 | 0.005 | 0.002 | 0.002 | 0.002 | 0.001 | 0.001 | 0.001 |
| Proportion explained | 0.092 | 0.064 | 0.043 | 0.022 | 0.018 | 0.015 | 0.011 | 0.010 | 0.007 |
| Cumulative proportion | 0.778 | 0.842 | 0.886 | 0.907 | 0.925 | 0.940 | 0.951 | 0.961 | 0.969 |
|  |  |  |  |  |  |  |  |  |  |
|  | PC10 | PC11 | PC12 | PC13 | PC14 | PC15 | PC16 | PC17 |  |
| Eigenvalue | 0.001 | 0.001 | 0.001 | 0.000 | 0.000 | 0.000 | 0.000 | 0.000 |  |
| Proportion explained | 0.007 | 0.006 | 0.005 | 0.004 | 0.004 | 0.003 | 0.002 | 0.001 |  |
| Cumulative proportion | 0.975 | 0.981 | 0.987 | 0.991 | 0.995 | 0.997 | 0.999 | 1.000 |  |
|  |  |  |  |  |  |  |  |  |  |
| **Accumulated constrained eigenvalues:** | | | |  |  |  |  |  |  |
|  | RDA1 | RDA2 | RDA3 | RDA4 | RDA5 | RDA6 | RDA7 | RDA8 |  |
| Eigenvalue | 0.054 | 0.013 | 0.005 | 0.002 | 0.001 | 0.001 | 0.000 | 0.000 |  |
| Proportion explained | 0.710 | 0.171 | 0.061 | 0.033 | 0.012 | 0.007 | 0.005 | 0.002 |  |
| Cumulative proportion | 0.710 | 0.880 | 0.941 | 0.974 | 0.986 | 0.993 | 0.998 | 1.000 |  |
|  |  |  |  |  |  |  |  |  |  |
| **Biplot scores for constraining variables:** | | | |  |  |  |  |  |  |
|  | RDA1 | RDA2 | RDA3 | RDA4 | RDA5 | RDA6 | RDA7 | RDA8 |  |
| Fire | 0.428 | 0.360 | 0.337 | -0.193 | -0.567 | -0.146 | -0.157 | -0.411 |  |
| Grazing | 0.182 | 0.406 | -0.036 | -0.012 | -0.226 | 0.784 | 0.197 | -0.310 |  |
| Temp | -0.824 | -0.351 | 0.118 | -0.173 | -0.256 | -0.003 | -0.213 | 0.207 |  |
| NAO | -0.119 | -0.318 | -0.515 | -0.670 | 0.261 | -0.241 | 0.041 | 0.205 |  |
| CuN | -0.918 | -0.032 | 0.148 | 0.257 | -0.231 | 0.071 | 0.052 | -0.081 |  |
| PC1.Anthro | -0.819 | 0.546 | 0.002 | 0.158 | 0.015 | -0.016 | -0.068 | 0.000 |  |
| Pop | -0.845 | 0.457 | 0.023 | 0.073 | -0.188 | -0.002 | 0.037 | 0.189 |  |
| Area | 0.944 | -0.076 | -0.292 | -0.004 | 0.074 | -0.048 | -0.057 | -0.087 |  |
|  |  |  |  |  |  |  |  |  |  |
| **r-squared:** | 0.686 |  |  |  |  |  |  |  |  |
| **adj r-squared:** | 0.625 |  |  |  |  |  |  |  |  |
|  | | |  |  |  |  |  |  |  |
|  | Fire | Grazing | Temp | NAO | CuN | PC1.Anthro | Pop | Area |  |
| **Variance Inflation Factors:** | 1.626 | 1.505 | 8.544 | 2.708 | 11.672 | 14.963 | 16.593 | 13.826 |  |
|  |  |  |  |  |  |  |  |  |  |
| **Significance of model:** | | |  |  |  |  |  |  |  |
|  | Df | Variance | F | Pr(>F) |  |  |  |  |  |
| Model | 8 | 0.076 | 11.209 | 1.00E-05 | *** |  |  |  |  |
| Residual | 41 | 0.035 |  |  |  |  |  |  |  |
|  |  |  |  |  |  |  |  |  |  |
| **Significance of axes:** | |  |  |  |  |  |  |  |  |
|  | Df | Variance | F | Pr(>F) |  |  |  |  |  |
| RDA1 | 1 | 0.054 | 63.632 | 1.00E-05 | *** |  |  |  |  |
| RDA2 | 1 | 0.013 | 15.307 | 1.00E-05 | *** |  |  |  |  |
| RDA3 | 1 | 0.005 | 5.454 | 0.005 | ** |  |  |  |  |
| RDA4 | 1 | 0.002 | 2.955 | 0.215 |  |  |  |  |  |
| RDA5 | 1 | 0.001 | 1.046 | 0.967 |  |  |  |  |  |
| RDA6 | 1 | 0.001 | 0.653 | 0.995 |  |  |  |  |  |
| RDA7 | 1 | 0.000 | 0.435 | 0.996 |  |  |  |  |  |
| RDA8 | 1 | 0.000 | 0.190 | 0.998 |  |  |  |  |  |
| Residual | 41 | 0.035 |  |  |  |  |  |  |  |
|  |  |  |  |  |  |  |  |  |  |
| **Significance of terms:** | | |  |  |  |  |  |  |  |
|  | Df | Variance | F | Pr(>F) |  |  |  |  |  |
| Fire | 1 | 0.012 | 14.609 | 1.00E-05 | *** |  |  |  |  |
| Grazing | 1 | 0.002 | 2.220 | 0.073 | . |  |  |  |  |
| Temp | 1 | 0.031 | 36.459 | 1.00E-05 | *** |  |  |  |  |
| NAO | 1 | 0.004 | 4.371 | 0.006 | ** |  |  |  |  |
| CuN | 1 | 0.013 | 15.717 | 1.00E-05 | *** |  |  |  |  |
| PC1.Anthro | 1 | 0.010 | 11.347 | 5.00E-05 | *** |  |  |  |  |
| Pop | 1 | 0.001 | 1.299 | 0.245 |  |  |  |  |  |
| Area | 1 | 0.003 | 3.818 | 0.014 | * |  |  |  |  |
| Residual | 41 | 0.035 |  |  |  |  |  |  |  |
| Signif. codes: 0 ‘***’ 0.001 ‘**’ 0.01 ‘*’ 0.05 ‘.’ 0.1 ‘ ’ 1 | | | | | |  |  |  |  |

Supplementary Table 4. Forward selection procedure for RDA_forward_.

| **Step** | **Explanatory variables** | **Df** | **AIC** | **F** | **Pr(>F)** | **Significance** | **R^2^_adj_** |
| --- | --- | --- | --- | --- | --- | --- | --- |
| 0 | None |  |  |  |  |  | 0.000 |
| 1 | Area | 1 | -136.110 | 37.407 | 1.00E-05 | *** | 0.426 |
| 2 | Area + PC1.Anthro | 1 | -143.160 | 9.324 | 1.00E-05 | *** | 0.511 |
| 3 | Area + PC1.Anthro + NAO | 1 | -146.730 | 5.421 | 3.00E-05 | *** | 0.553 |
| 4 | Area + PC1.Anthro + NAO + cuN | 1 | -152.780 | 7.863 | 1.00E-05 | * | 0.611 |
| 5 | Area + PC1.Anthro + NAO + cuN + Fire | 1 | -153.110 | 2.097 | 0.044 | * | 0.620 |
| Signif. codes: 0 ‘***’ 0.001 ‘**’ 0.01 ‘*’ 0.05 ‘.’ 0.1 ‘ ’ 1 | | | | | | | |

**Supplementary Table 5**. Model output and significance testing for RDA_forward_.

| **Call: rda(formula = NAP ~ Area + PC1.Anthro + NAO + CuN + Fire, data = Env.all)** | | | | | | | | | |
| --- | --- | --- | --- | --- | --- | --- | --- | --- | --- |
| **Partitioning of variance:** | | |  |  |  |  |  |  |  |
|  | Variance | Proportion |  |  |  |  |  |  |  |
| Total | 0.110 | 1.000 |  |  |  |  |  |  |  |
| Constrained | 0.073 | 0.659 |  |  |  |  |  |  |  |
| Unconstrained | 0.038 | 0.341 |  |  |  |  |  |  |  |
|  |  |  |  |  |  |  |  |  |  |
| **Eigenvalues and their contribution to the variance:** | | | | | | | | | |
|  | RDA1 | RDA2 | RDA3 | RDA4 | RDA5 |  |  |  |  |
| Eigenvalue | 0.053 | 0.012 | 0.004 | 0.002 | 0.001 |  |  |  |  |
| Proportion explained | 0.483 | 0.111 | 0.039 | 0.021 | 0.006 |  |  |  |  |
| Cumulative proportion | 0.483 | 0.594 | 0.633 | 0.654 | 0.659 |  |  |  |  |
|  |  |  |  |  |  |  |  |  |  |
|  | PC1 | PC2 | PC3 | PC4 | PC5 | PC6 | PC7 | PC8 | PC9 |
| Eigenvalue | 0.011 | 0.008 | 0.005 | 0.003 | 0.002 | 0.002 | 0.001 | 0.001 | 0.001 |
| Proportion explained | 0.098 | 0.071 | 0.044 | 0.023 | 0.020 | 0.019 | 0.013 | 0.011 | 0.008 |
| Cumulative proportion | 0.757 | 0.828 | 0.872 | 0.895 | 0.914 | 0.933 | 0.946 | 0.957 | 0.965 |
|  |  |  |  |  |  |  |  |  |  |
|  | PC10 | PC11 | PC12 | PC13 | PC14 | PC15 | PC16 | PC17 |  |
| Eigenvalue | 0.001 | 0.001 | 0.001 | 0.001 | 0.000 | 0.000 | 0.000 | 0.000 |  |
| Proportion explained | 0.007 | 0.007 | 0.006 | 0.005 | 0.004 | 0.003 | 0.002 | 0.001 |  |
| Cumulative proportion | 0.972 | 0.979 | 0.985 | 0.990 | 0.994 | 0.997 | 0.999 | 1.000 |  |
|  |  |  |  |  |  |  |  |  |  |
| **Accumulated constrained eigenvalues:** | | | | | | | | | |
|  | RDA1 | RDA2 | RDA3 | RDA4 | RDA5 |  |  |  |  |
| Eigenvalue | 0.053 | 0.012 | 0.004 | 0.002 | 0.001 |  |  |  |  |
| Proportion explained | 0.733 | 0.168 | 0.059 | 0.032 | 0.008 |  |  |  |  |
| Cumulative proportion | 0.733 | 0.901 | 0.960 | 0.992 | 1.000 |  |  |  |  |
|  |  |  |  |  |  |  |  |  |  |
| **Biplot scores for constraining variables:** | | | | | | | | | |
|  | RDA1 | RDA2 | RDA3 | RDA4 | RDA5 |  |  |  |  |
| Area | 0.947 | -0.066 | -0.312 | 0.024 | -0.012 |  |  |  |  |
| PC1.Anthro | -0.825 | 0.547 | -0.035 | -0.139 | -0.002 |  |  |  |  |
| NAO | -0.118 | -0.346 | -0.481 | 0.742 | 0.289 |  |  |  |  |
| CuN | -0.922 | -0.047 | 0.164 | -0.278 | -0.211 |  |  |  |  |
| Fire | 0.428 | 0.385 | 0.336 | 0.191 | -0.721 |  |  |  |  |
|  |  |  |  |  |  |  |  |  |  |
| **r-squared:** | 0.659 |  |  |  |  |  |  |  |  |
| **adj r-squared:** | 0.620 |  |  |  |  |  |  |  |  |
|  |  |  |  |  |  |  |  |  |  |
|  | Area | PC1.Anthro | NAO | CuN | Fire |  |  |  |  |
| **Variance Inflation Factors:** | 9.810 | 3.031 | 1.603 | 8.535 | 1.428 |  |  |  |  |
|  |  |  |  |  |  |  |  |  |  |
| **Significance of model:** | | | | | | | | | |
|  | Df | Variance | F | Pr(>F) |  |  |  |  |  |
| Model | 5 | 0.073 | 17.015 | 1.00E-05 | *** |  |  |  |  |
| Residual | 44 | 0.038 |  |  |  |  |  |  |  |
|  |  |  |  |  |  |  |  |  |  |
| **Significance of axes:** | | | | | | | | | |
|  | Df | Variance | F | Pr(>F) |  |  |  |  |  |
| RDA1 | 1 | 0.053 | 62.393 | 1.00E-05 | *** |  |  |  |  |
| RDA2 | 1 | 0.012 | 14.264 | 1.00E-05 | *** |  |  |  |  |
| RDA3 | 1 | 0.004 | 5.016 | 0.001 | *** |  |  |  |  |
| RDA4 | 1 | 0.002 | 2.680 | 0.045 | * |  |  |  |  |
| RDA5 | 1 | 0.001 | 0.720 | 0.662 |  |  |  |  |  |
| Residual | 44 | 0.038 |  |  |  |  |  |  |  |
|  |  |  |  |  |  |  |  |  |  |
| **Significance of terms:** | | | | | | | | | |
|  | Df | Variance | F | Pr(>F) |  |  |  |  |  |
| Area | 1 | 0.048 | 56.532 | 1.00E-05 | *** |  |  |  |  |
| PC1.Anthro | 1 | 0.010 | 12.008 | 4.00E-05 | *** |  |  |  |  |
| NAO | 1 | 0.005 | 6.382 | 0.001 | *** |  |  |  |  |
| CuN | 1 | 0.007 | 8.055 | 0.000 | *** |  |  |  |  |
| Fire | 1 | 0.002 | 2.097 | 0.085 | . |  |  |  |  |
| Signif. codes: 0 ‘***’ 0.001 ‘**’ 0.01 ‘*’ 0.05 ‘.’ 0.1 ‘ ’ 1 | | | | | | | | | |
